# Species richness and taxonomic composition of trawl macrofauna of the North Pacific and its adjacent seas

**DOI:** 10.1038/s41598-018-34819-4

**Published:** 2018-11-09

**Authors:** Igor V. Volvenko, Alexei M. Orlov, Andrey V. Gebruk, Oleg N. Katugin, Georgy M. Vinogradov, Olga A. Maznikova

**Affiliations:** 1 Pacific Research Fisheries Center (TINRO-Center), Vladivostok, 690091 Russia; 2 Russian Federal Research Institute of Fisheries and Oceanography (VNIRO), Moscow, 107140 Russia; 3 A.N. Severtsov Institute of Ecology and Evolution, Russian Academy of Sciences (IPEE), Moscow, 119071 Russia; 4 Dagestan State University (DSU), Makhachkala, 367000 Russia; 5 Tomsk State University (TSU), Tomsk, 634050 Russia; 6 Caspian Institute of Biological Resources, Dagestan Scientific Center, Russian Academy of Sciences (CIBR DSC RAS), Makhachkala, 367023 Russia; 7 P.P. Shirshov Institute of Oceanology, Russian Academy of Sciences (IO RAS), Moscow, 117997 Russia

## Abstract

A checklist is presented of animal species obtained in 68,903 trawl tows during 459 research surveys performed by the Pacific Research Fisheries Center (TINRO-Center) over an area measuring nearly 25 million km^2^ in the Chukchi and Bering seas, Sea of Okhotsk, Sea of Japan and North Pacific Ocean in 1977–2014 at depths of 5 to 2,200 m. The checklist comprises 949 fish species, 588 invertebrate species, and four cyclostome species (some specimens were identified only to genus or family level). For each species details are given on the type of trawl (benthic and/or pelagic) and basins where the species was found. Comprehensiveness of data, taxonomic composition of catches, dependence of species richness on the survey area, sample size, and habitat, are considered. Ratios of various taxonomic groups of trawl macrofauna in pelagic and benthic zones and in different basins are analysed. Basins are compared based on species composition.

## Introduction

**Figure 1:**
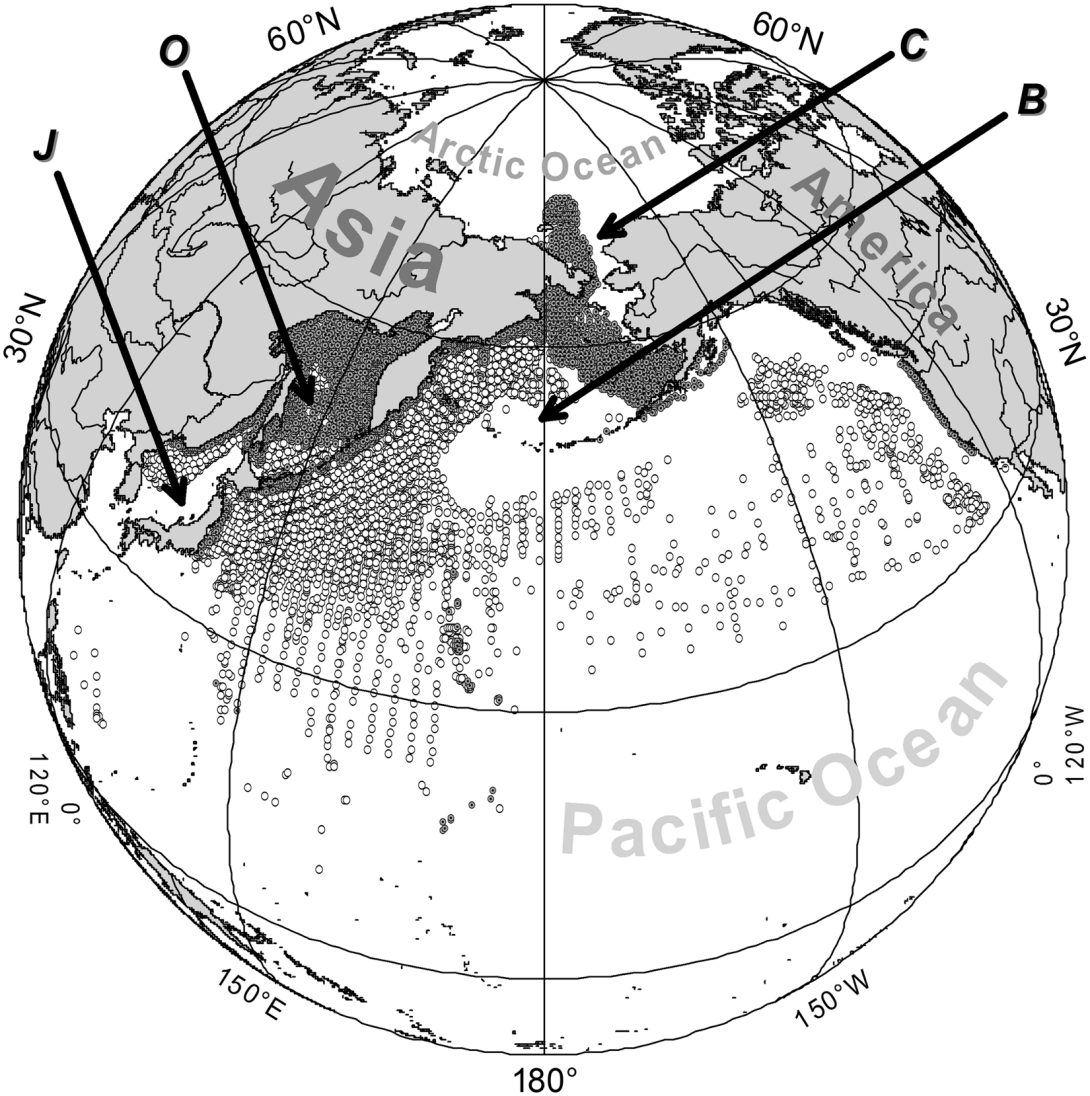
Spatial distribution of midwater (open circles) and bottom (dark circles) trawl stations used to compile the trawl macrofauna checklist. Key to basins (as in text): B, Bering Sea; C, Chukchi Sea; J, Sea of Japan; O, Sea of Okhotsk; P, Pacific Ocean.

The region where material for the present study was collected (Fig. 1) is one of the most productive and economically important regions in the World Ocean^1–6^. It includes the Chukchi and Bering seas, Sea of Okhotsk, Sea of Japan, and North Pacific Ocean, and provides more than 2/3 of Russian fish catches^7–10^ and a large part of the catches of Canada, China, Japan, South Korea and the USA^11–17^.

In accordance with the principles of sustainable use of natural resources, based on the ecosystem approach to their study and management (see for example^18–22^), monitoring of marine communities and their environment has been carried out in the study region for many years. Most large-scale multi-purpose marine expeditions to the area have been conducted by the Federal State Budgetary Scientific Establishment “Pacific Research Fisheries Center” (TINRO-Center)^23^. Records of nekton, benthos and macroplankton (the latter includes large jellyfishes, comb jellies, pelagic tunicates, etc.) in these expeditions are based on trawl catches. The bulk of information from these surveys in the TINRO-Center Regional Data Center^24^ relates to “trawl macrofauna”. Under this term we consider animals with a body size from 1 cm to several meters weighing from several grams to hundreds of kilograms caught by bottom and midwater trawls with a fine-mesh liner in the cod end. The present paper is based on data obtained using such gear types.

The main objective of the present study is to produce a species checklist of fishes, cyclostomes and invertebrates recorded during TINRO-Center trawl surveys in the North Pacific and adjacent Arctic regions (Chukchi Sea) over a period of 38 years. Each species entry provides the information on basin(s) where the species was collected, and trawl type (bottom or midwater).

Also, we present a brief analysis of the checklist, examining current knowledge of regional trawl macrofauna in the regions considered, its taxonomic composition, species richness in different areas, dependence on survey area and sample size, and habitat. We also compared species richness and taxonomic composition among the basins.

## Materials and Methods

### Sources of data

Information was obtained mainly from two large databases^25,26^ supplemented by materials from trawl surveys conducted until 2014. These surveys were conducted in accordance with the programs approved by the TINRO-Center management and agreed with the Russian Federation Ministry of Agriculture Federal Agency for Fisheries. The sampling area (Table 1) covers nearly 25 million km^2^. Specimens were collected at 36,640 bottom trawl stations in depths of five to 2,000 m, and at 32,263 mid-water trawl stations mostly at depths from the sea surface (0 m) down to 1,000 m, although some mesopelagic hauls reached 2,200 m. Both types of trawls (bottom and midwater) were supplied with a 10–12-mm fine-mesh liner in the cod end. Almost one billion individuals of various macrofauna species have been recorded in the trawl catches. Whenever possible, all taxa have been identified to species level.

**Table 1:** Parameters of samples used to generate the checklist.

Basin	Zone	Survey years	Depth range, m	Number of stations	Study area, thousand km^2^	Total trawling time, hours	Total sampling area, km^2^	Number of individuals sampled
Chukchi Sea	Pelagic	2003–2014	0–91	239	298	162	40	1,701,314
	Benthic	1995–2014	13–222	237	286	118	10	631,531
	Combined	1995–2014	0–222	476	298	280	50	2,332,845
Bering Sea	Pelagic	1982–2014	0–920	4,959	1,419	5,939	1,966	68,718,728
	Benthic	1977–2014	6–1,400	9,235	1,028	6,608	901	23,978,418
	Combined	1977–2014	0–1,400	14,194	2,126	12,547	2,867	92,697,146
Sea of Okhotsk	Pelagic	1980–2014	0–1,000(2,200)	11,053	1,523	10,598	3,232	98,376,567
	Benthic	1977–2014	5–2,000	10,073	1,385	7,159	819	33,190,559
	Combined	1977–2014	0–2,200	21,126	1,523	17,757	4,051	131,567,126
Sea of Japan	Pelagic	1981–2013	0–720	2,621	447	2,456	836	34,66, 510
	Benthic	1978–2014	5–935	10,766	137	6,235	591	13,59, 004
	Combined	1978–2014	0–935	13,387	447	8,691	1,428	48,256,514
Pacific Ocean	Pelagic	1979–2014	0–1,000(1,230)	13,391	17,741	19,859	7,720	538,822,020
	Benthic	1977–2012	10–1,860	6,329	1,262	8,150	1,498	34,732,062
	Combined	1977–2014	0–1,860	19,720	20,236	28,009	9,217	573,554,082
Total area	Pelagic	1979–2014	0–2,200	32,263	21,429	39,014	13,794	742,282,139
	Benthic	1977–2014	5–2,000	36,640	4,097	28,271	3,819	106,125,574
	Combined	1977–2014	0–2,200	68,903	24,630	67,285	17,613	848,407,713

Maximum depth (at which only few trawls were taken) is shown in parentheses; study area includes all trawl stations and was calculated by contouring areas with stations (see Fig. 1); total sampling area was calculated as the sum of areas covered by trawl hauls; area of a trawl haul was calculated by multiplying trawl horizontal opening by trawling distance.

This study does not include information from commercial fisheries, and is based only on reliable information from 459 selected research cruises, where data were obtained by skilled ichthyologists and hydrobiologists. In these cruises, unidentified specimens were preserved and delivered to onshore laboratories for further identification by experts in zoological taxonomy. However, this does not mean that all species identifications were correct, especially in groups with difficult and complex higher taxa, such as the fish families Myctophidae, Liparidae and Zoarcidae. Therefore, data obtained from the databases were further scrutinised by taxonomic experts.

### Verification of data

Where species occurrence is considered ambiguous, information (coordinates, depth, time, catch size, and size of individuals) was analysed further and compared with published data. If an individual of a species was found too far outside the known species range, it was excluded from the list. In such cases, the specimen was referred to a higher taxon (genus or family, as considered appropriate). Conversely, in cases when an animal was identified to genus or family level, and only a single species of this higher taxon is known to occur in a region (based on published data), the animal was identified as that species. A number of records were excluded as unreliable and incorrigible if no specimens had been photographed, deposited in a museum or sampled for genetic analysis. For example, several catches of the frilled shark *Chlamydoselachus anguineus* in the Sea of Okhotsk were excluded: according to published data, this species does not occur in the Sea of Okhotsk and cannot be confused with other species. In some cases, species identifications were corrected to conform with accepted valid senior synonyms. In total, corrections were made for 267 fish species, 33 cephalopod species and 99 species of other invertebrates.

At the intermediate stage, the checklist included the verified species list and the list of animals not identified to species level. From the latter list, we selected those genera and families absent from the verified species list, and added them to that list, since each such entry corresponds to at least one species not included in the main checklist because of incomplete specimen identification. Since some of these records may correspond to more than one species, the total length of the checklist with 1,541 entries reflects the lower limit of potential species richness of the trawl macrofauna in the surveyed area, which is therefore best expressed as “*at least* 1,541 species”. This protocol applies to species numbers on the seafloor, in the pelagic zone, in different basins (seas and ocean) and in different taxonomic groups. Note that the beard worms, phylum Pogonophora (currently referred to as the annelid family Siboglinidae) were never identified in trawl catches even to family level, so they are represented in the final checklist as a single entry.

Published and Internet data were used to further extend the accuracy of information on the presence (+) or absence (−) of a species in trawl catches in each specific basin by adding reliable species records not listed in the TINRO-Center databases. In these cases, a species that occurs in a basin but did not occur in our samples is marked with an asterisk (*). Therefore, in the final checklist, only species definitely absent from a particular basin, based both on our data and published data, are marked with as absent (−).

For example, the large sea lily *Heliometra glacialis* occurs in all surveyed basins and was common in bottom trawl catches but it was absent from the Chukchi Sea samples. Therefore, in the checklist, this species is marked with an asterisk for the Chukchi Sea and a “+” for all other marine regions. The bivalve *Pododesmus macrochisma* also occurred in bottom trawls and was present in catches from open ocean areas, the Sea of Japan and Okhotsk Sea, and so was marked “+” for those marine regions. It is also known from the Bering Sea, but was not recorded in our trawl samples there, so for that basin it is marked with an asterisk. This species is not known from the Chukchi Sea and it was not recorded from trawl catches so for that basin it was marked as absent.

### Data restrictions

No attempt was made to generate a complete list of all the fauna from the area shown in Fig. 1. In the checklist, there are no species that did not occur in trawl catches but were taken by grabs, hydraulic dredges, longlines, traps, divers, etc. Therefore, the checklist is named “the trawl macrofauna”, since it includes only the animals that were caught by trawl in the surveyed area.

The minimum depth of bottom hauls in this study ranged from five to 13 m depending on the size of the trawler, its draught, trawl construction and operational characters, such as the smallest vertical opening. Most pelagic hauls (including surface trawls at 0 m depth) were made in areas deeper than 25–30 m, corresponding to the minimum vertical opening of the majority of Russian midwater trawls. Consequently, coastal zone fauna is weakly represented, since many inhabitants of the littoral and upper sublittoral zones were not encountered and only species from its outer periphery are included. That is why, for example, the common commercial clam *Ruditapes philippinarum*, which occurs in coastal habitats at depths 0.5–4 m protected from strong surf, is not included in the checklist. This is a burrowing mollusk with main populations living at 1–3 m depth in sandy or gravel-pebble sediments^27^.

### Additional data sources

To verify information on geographical distribution, taxonomic status, and accepted scientific species names, we used 54 publications^28–81^ and 42 online resources (Table 2). For fishes and cyclostomes, we relied on the following websites: Eschmeyer W.N., Fricke R., van der Laan R. editors (2018) “Catalog of fishes: genera, species, references”; and Froese R., Pauly D. editors (2018) “FishBase” (No. 22 and 28 in Table 2). For invertebrates, we used WoRMS Editorial Board (2018) “World Register of Marine Species” (No. 34 in Table 2). We consider these sources of information as the most reliable professional modern knowledge bases. However, in some cases, where other authors convincingly argue in favour of other species names or ranges, such alternatives were accepted.

**Table 2:** List of URLs providing information on taxonomy and species geographic distribution.

No	URL (Uniform Resource Locator)
1	http://arctos.database.museum/taxonomy.cfm
2	http://argus.aqualogo.ru
3	http://bie.ala.org.au
4	http://bryozone.myspecies.info
5	http://clade.ansp.org/obis/find_mollusk.html
6	http://collections.nmnh.si.edu/search/iz
7	http://collections.peabody.yale.edu/search/Search
8	http://eol.org
9	http://fauna-flora.ru
10	http://fish.dvo.ru
11	http://glgolub.narod2.ru
12	http://ispecies.org
13	http://marinebio.org
14	http://polychaetes.lifewatchgreece.eu
15	http://ribovodstvo.com/books/item/f00/s00/z0000004/index.shtml
16	http://shark-references.com
17	http://species-identification.org
18	http://tolweb.org
19	http://www.annelida.net
20	http://www.arcodiv.org
21	http://www.bagniliggia.it/WMSD/WMSDsearch.htm
22	http://www.calacademy.org/scientists/projects/catalog-of-fishes
23	http://www.catalogueoflife.org
24	http://www.conchology.be
25	http://www.crabs.ru/
26	http://www.fao.org/figis/geoserver/factsheets/species.html
27	http://www.fegi.ru/primorye/atlas
28	http://www.fishbase.org
29	http://www.gastropods.com
30	http://www.gbif.org
31	http://www.inaturalist.org
32	http://www.itis.gov
33	http://www.iucnredlist.org
34	http://www.marinespecies.org
35	http://www.marlin.ac.uk/biotic/
36	http://www.octe.ru
37	http://www.sealifebase.org
38	http://www.species-identification.org
39	http://www.ubio.org
40	http://www.zin.ru/zoodiv
41	https://en.wikipedia.org
42	https://ru.wikipedia.org

Web-sites are given in alphabetical order of URLs; date of the latest access to all sites was July 10, 2018.

### Statistical methods

Relationships between the ratio of species missed by trawl surveys and the number of discovered species to the size of the surveyed area and other parameters were investigated by regression analysis using the method of least squares, with the use, where necessary, of linearizing transformations of variables^82,83^.

Comparisons among basins by species composition using cluster analysis^84–88^ were performed using three different algorithms: (1) single linkage (SL) or nearest neighbour, when clusters are joined based on the smallest distance between the two groups; (2) unweighted pair-group average (UPA), when clusters are joined based on the average distance between all members in the two groups; and (3) Ward’s method, when clusters are joined in a way that the increase in within-group variance is minimized. Also, 13 measures of similarity were used based on binary (presence-absence) data listed in the section on comparison of basins by species composition. The following symbols are used traditionally in this approach (ibid): *a*, number of species present in both of the compared lists; *b*, number of species present in the second list but missing from the first list; *c*, the number of species present in the first list but missing in the second list; and *d*, the number of species missing from both lists, but present in other lists with the total number of *S* species. Combinations of symbols: *a* + *c*, number of species present in the first list; *a* + *b*, the number of species present in the second list; *b* + *d*, number of species missing from the first list; *c* + *d*, number of species missing from the second list; *a* + *b* + *c* + *d* = *S*, total number of species; *a* + *b* + *c* = *S* − *d*, number of species present in at least one of the two lists. When pairs of lists are compared: *a* corresponds to the number of positive matches; *d*, the number of negative matches; *a* + *d*, the number of positive and negative matches; *b* + *c*, the number of mismatches of either kind.

Subsequently, measures of similarity were subdivided into two groups: (1) similarity coefficients that treat *a* and *d* symmetrically, taking into account both species presence and absence (i.e., the number of positive and negative matches: we used five of such measures); (2) coefficients that take into account only species presence and ignoring the number of negative matches (the value of *d*: we used eight such measures). The same measures of similarity were used for comparison of species lists by alternative non-hierarchical methods of multivariate analyses: metric and non-metric multidimensional scaling^89^.

## Results and Discussion

### The checklist

The list compiled is presented in Supplementary Table. It includes 1,541 lines (corresponding to our minimum estimate of the trawl macrofauna species richness in the study area) and 10 columns. The first column shows the scientific name of a species (genus, family) in alphabetical order: to simplify the use of the table by non-experts in taxonomy, they are not arranged by taxa. This enables users to quickly find the scientific name of a species of interest without requiring detailed knowledge of its taxonomy.

The second column “Taxon” is a numeric code, corresponding to one of 20 aggregate higher taxonomic groups:

1. Fishes;
2. Cyclostomes (lampreys and hagfishes);
3. Ascidians and pelagic tunicates (salps and appendicularians);
4. Crabs (Brachyura) and craboids (lithodids from Anomura);
5. Shrimps and crangonids;
6. Other crustaceans (hermit-crabs, burrowing mantis shrimps, squat lobsters, isopods, amphipods, and cirripeds);
7. Cephalopods (paper nautiluses, octopuses, squids, and cuttlefishes);
8. Gastropods including pelagic ones (heteropods, pteropods, and nudibranchs);
9. Bivalves;
10. Other molluscs – polyplacophorans (chitons) and solenogasters;
11. Sea urchins;
12. Sea cucumbers;
13. Other echinoderms (brittle stars, starfishes and sea lilies);
14. Coelenterates (jelly-fishes, polyps, corals, sea fans, and anemones);
15. Comb jellies;
16. Bryozoans;
17. Sponges;
18. Pycnogonids (pantopods or sea spiders);
19. Brachiopods;
20. The final group contains miscellaneous “Other invertebrates” (annelid polychaetes, flat worms, nemerteans, sipunculans, priapulans, pogonophorans); that is, an aggregate group (mainly “worms”) rarely found in trawls and lacking in commercial value.

The third column, “Gear”, notes occurrence in midwater (pelagic) and/or bottom trawl catches. Columns four to eight indicate occurrence in each of five basins: the Bering Sea (B), Chukchi Sea (C), Sea of Japan (J), Sea of Okhotsk (O), Pacific Ocean (P). Presence of a species is indicated by “+”, absence by “−”, and “*” means absence from catches but presence according to previously published data.

### Comprehensiveness of data and state of our knowledge of trawl macrofauna

Analysis of the comprehensiveness of databases reveals that the macrofauna is represented unevenly in TINRO-Centre trawl surveys (Table 3). In the Sea of Okhotsk and Sea of Japan, 15% and 18% of species, respectively, were absent from trawl surveys and were included in the checklist based on published data. The proportion of absent species is almost a quarter in the Pacific Ocean, slightly less than a third in the Bering Sea, and almost one half in the Chukchi Sea. There is an inverse relationship between these ratios and sample size in each basin (Table 4).

**Table 3:** Number (% in parentheses) of species in each basin and habitat based on Supplementary Table and additional sources.

Basin	Zone	Present in database, i.e. captured at trawl stations shown in Fig. 1 (symbol “+” in Supplementary Table)	Added from publications (symbol “*” in Supplementary Table)	Total
Chukchi Sea	Pelagic	79 (70)	34 (30)	113 (100)
	Benthic	153 (56)	121 (44)	274 (100)
	Combined	154 (55)	125 (45)	279 (100)
Bering Sea	Pelagic	292 (95)	14 (5)	306 (100)
	Benthic	491 (72)	187 (28)	678 (100)
	Combined	504 (72)	193 (28)	697 (100)
Sea of Okhotsk	Pelagic	357 (95)	18 (5)	375 (100)
	Benthic	700 (85)	124 (15)	824 (100)
	Combined	723 (85)	130 (15)	853 (100)
Sea of Japan	Pelagic	232 (88)	33 (12)	265 (100)
	Benthic	540 (84)	104 (16)	644 (100)
	Combined	555 (82)	123 (18)	678 (100)
Pacific Ocean	Pelagic	665 (95)	36 (5)	701 (100)
	Benthic	757 (72)	300 (28)	1,057 (100)
	Combined	1,032 (77)	310 (23)	1,342 (100)

**Table 4:** Pearson’s correlations between the share of the number of species in region that were not observed in TINRO-Center trawl surveys (from Table 3) and five sample parameters (from Table 1).

Sample parameter	Pelagic	Benthic	Total
Number of stations	**−0.941**	**−0.946**	**−0.925**
Study area (thousand km^2^)	−0.729	−0.288	−0.623
Total trawling time (hours)	**−0.946**	−0.848	**−0.911**
Total area covered by trawl hauls (km^2^)	**−0.948**	−0.846	**−0.910**
Number of individuals captured (millions)	**−0.949**	−0.843	**−0.908**

Linear, exponential, reciprocal, logarithmic, multiplicative, square root and other simple regression models were tested in the course of correlation analysis. Best models were selected by minimum residual variance and p-values, and maximum correlation coefficients. Coefficients with p-values < 0.05 are shown in bold.

The fauna of the pelagic zone is more completely represented in the surveys than the seafloor fauna. Only 5% of pelagic species were not captured in trawl nets in the Bering Sea, Sea of Okhotsk and Pacific Ocean, 12% in the Sea of Japan, and 30% in the Chukchi Sea. Non-capture proportions are higher for benthic species, ranging from 15% to 44% (Table 3).

As expected, despite the difference in numbers, the inverse relationship between the ratio of species missed by trawl surveys and the survey effort is true for both pelagic and benthic fauna and also for combined fauna (Table 4). Pelagic surveys show better comprehensiveness than benthic surveys at a smaller number of stations. However, other sample size features for the pelagic zone are greater than those for the seafloor (Table 1).

Different taxa are unevenly represented in databases (Table 5). The best represented are cyclostomes and fishes, since known species absent from the database account for only 0% and 5% of known species, respectively, not captured in at least one basin. Among invertebrates (41% of species absent from catches) the best represented are molluscs (<1/3 of known species absent), and the worst are sponges (>50% of species absent), polychaetes, and some rare benthic invertebrate macrofauna taxa. In part, this reflects failure to obtain them by the survey gear used, because of factors such as their small size, or a sessile or burrowing mode of life.

**Table 5:** Number (% in parentheses) of species identified from different taxonomic groups based on Supplementary Table, including species not present in the database at least from one basin.

Taxon/Group	Total number in the checklist	Including species found at least in one basin based only on published data (“*” in Supplementary Table)
Fish	949 (100)	48 (5)
Cyclostomes	4 (100)	0 (0)
Tunicates	21 (100)	10 (48)
Crustaceans	131 (100)	55 (42)
Mollusks	256 (100)	79 (31)
Echinoderms	85 (100)	38 (45)
Coelenterates	42 (100)	20 (48)
Sponges	15 (100)	11 (73)
Other invertebrates	38 (100)	28 (74)
Total invertebrates	588 (100)	241 (41)
Total macrofauna	1,541 (100)	289 (19)

To reveal the proportion of the trawl macrofauna in the whole marine fauna, total species numbers (“+” and “*” in Table 3) for each basin were compared with data published by Parin *et al*.^75^ for fishes and cyclostomes, including species at depths that were not covered by our surveys, and species that were not caught by trawls; and data published by Sirenko *et al*.^65^ for invertebrates, including littoral, deep-sea species, meso- and microfauna, plankton, infauna, etc. The macrofauna in the checklists corresponds to 10% of all fauna (including fish, cyclostomes and invertebrates) in the Chukchi Sea; 19% in the Bering Sea; 22% in the Sea of Okhotsk; 12% in the Sea of Japan; and 23% in the Pacific Ocean. There is a direct relationship between these proportions and sample sizes (Table 1).

Accepting that, within the entire study area, there exist four species of cyclostome^75^, 1,455 fish species^44^ and 6,771 macrobenthic species^65^, it appears that the trawl macrofauna covers all cyclostomes, 65% of fish species and only 11% of invertebrates (Table 5 second column). Some 23% of all macrofauna species (1,541 out of 6,771) can be considered as the “trawl macrofauna”.

Another concern related to the comprehensiveness of the checklist is the accuracy of macrofaunal taxonomic identifications. Table 6 indicates that 94% of animals that occurred in trawl hauls were identified to species, 4% to genus and 2% to family. However, these figures differ significantly for different taxonomic groups, depending on species richness and difficulty of identification.

**Table 6:** Number (% in parentheses) of species from Supplementary Table identified to different taxonomic levels.

Taxon/Group	Level of identification			Total number of species (100%)
	species	genus	family	
Fishes	910 (96)	26 (3)	13 (1)	949
Cyclostomes	4 (100)	0 (0)	0 (0)	4
Tunicates	14 (67)	6 (29)	1 (5)	21
Crabs and craboids	34 (94)	1 (3)	1 (3)	36
Shrimps	69 (99)	0 (0)	1 (1)	70
Other crustaceans	22 (88)	2 (8)	1 (4)	25
Cephalopods	82 (96)	3 (4)	0 (0)	85
Gastropods	103 (94)	5 (5)	1 (1)	109
Bivalves	53 (93)	4 (7)	0 (0)	57
Other mollusks	4 (80)	1 (20)	0 (0)	5
Sea urchins	8 (100)	0 (0)	0 (0)	8
Sea cucumbers	15 (94)	1 (6)	0 (0)	16
Other echinoderms	60 (98)	0 (0)	1 (2)	61
Coelenterates	34 (81)	6 (14)	2 (5)	42
Comb-jellies	3(100)	0 (0)	0 (0)	3
Bryozoans	7 (88)	0 (0)	1 (13)	8
Sponges	15 (100)	0 (0)	0 (0)	15
Pycnogonids	1 (100)	0 (0)	0 (0)	1
Brachiopods	1 (100)	0 (0)	0 (0)	1
Other benthic invertebrates	18 (72)	4 (16)	3 (12)	25
All invertebrates	543 (92)	33 (6)	12 (2)	588
Total macrofauna	1,457 (94)	59 (4)	25 (2)	1,541

### Taxonomic composition of fauna

**Figure 2:**
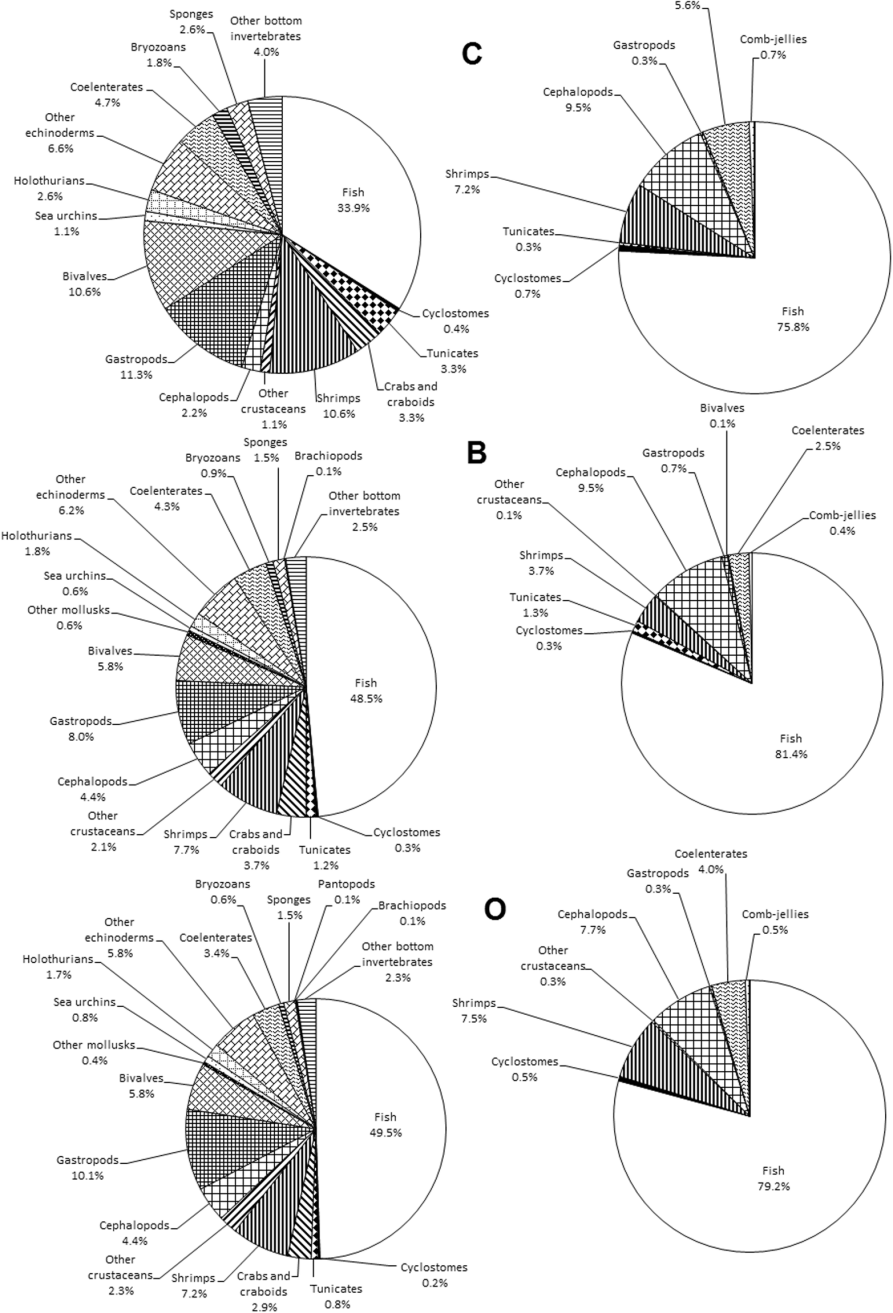
Percentage of species of different taxonomic groups in benthic (left diagrams) and pelagic (right diagrams) trawl catches from different seas (Key as in text and legend to Fig. 1).

**Figure 3:**
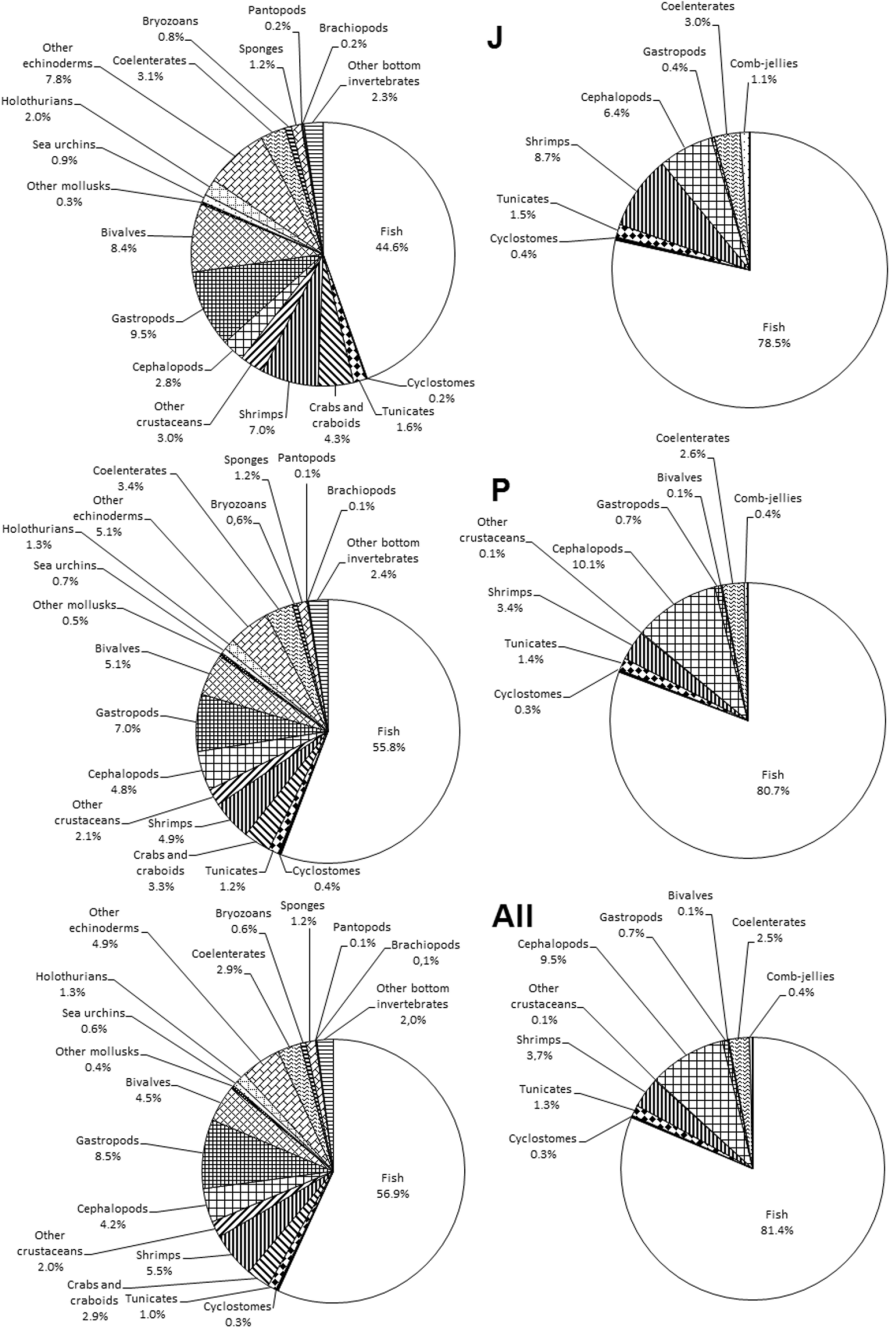
Percentage of species of different taxonomic groups in benthic (left diagrams) and pelagic (right diagrams) trawl catches from different marine regions (All, complete survey area; key to other marine regions as in text and legend to Fig. 1).

The following patterns have been revealed from species richness distribution by taxa (Table 7; Figs 2 and 3): First, there are more fish and cephalopod species in the pelagic zone than on the seabed, but all other groups are more speciose at the seabed, and some are completely absent from the pelagic zone. Second, the percentage of invertebrates is much higher in the Chukchi Sea and lower in the Pacific Ocean, compared to other basins.

**Table 7:** Species richness in different basins and habitats.

Taxon/Group	Total species Nr	Pelagic trawls	Bottom trawls	Chukchi Sea			Bering Sea			Sea of Okhotsk			Sea of Japan			Pacific Ocean		
				pelagic	benthic	total	pelagic	benthic	total	pelagic	benthic	total	pelagic	benthic	total	pelagic	benthic	total
Fish	949	611	709	78	93	93	232	329	340	297	408	431	208	287	312	566	590	821
Cyclostomes	4	2	4	1	1	1	2	2	2	2	2	2	1	1	1	2	4	4
Tunicates	21	10	13	0	9	9	1	8	9	0	7	7	4	10	13	10	13	21
Crabs and craboids	36	0	36	0	9	9	0	25	25	0	24	24	0	28	28	0	35	35
Shrimps	70	28	69	16	29	29	22	52	52	28	59	60	23	45	45	24	52	53
Other crustaceans	25	1	25	0	3	3	0	14	14	1	19	19	0	19	19	1	22	22
Cephalopods	85	71	52	4	6	6	29	30	31	29	36	36	17	18	20	71	51	84
Gastropods	109	5	106	0	31	31	1	54	54	1	83	83	1	61	61	5	74	77
Bivalves	57	1	56	0	29	29	0	39	39	0	48	48	0	54	54	1	54	55
Other mollusks	5	0	5	0	0	0	0	4	4	0	3	3	0	2	2	0	5	5
Sea urchins	8	0	8	0	3	3	0	4	4	0	7	7	0	6	6	0	7	7
Holothurians	16	0	16	0	7	7	0	12	12	0	14	14	0	13	13	0	14	14
Other echinoderms	61	0	61	0	18	18	0	42	42	0	48	48	0	50	50	0	54	54
Coelenterates	42	19	36	12	13	16	17	29	33	15	28	31	8	20	21	18	36	41
Comb-jellies	3	3	0	2	0	2	2	0	2	2	0	2	3	0	3	3	0	3
Bryozoans	8	0	8	0	5	5	0	6	6	0	5	5	0	5	5	0	6	6
Sponges	15	0	15	0	7	7	0	10	10	0	12	12	0	8	8	0	13	13
Pycnogonids	1	0	1	0	0	0	0	0	0	0	1	1	0	1	1	0	1	1
Brachiopods	1	0	1	0	0	0	0	1	1	0	1	1	0	1	1	0	1	1
Other benthic invertebrates	25	0	25	0	11	11	0	17	17	0	19	19	0	15	15	0	25	25
All invertebrates	588	138	533	34	180	185	72	347	355	76	414	420	56	356	365	133	463	517
Total macrofauna	1,541	751	1,246	113	274	279	306	678	697	375	824	853	265	644	678	701	1,057	1,342

The first pattern is commonplace; the second stems from the fact that, in the relatively shallow Chukchi Sea, the pelagic zone and the seabed were almost equally studied. On the other hand, in the Pacific Ocean, mainly narrow shelf and seamount summits have been surveyed using bottom trawls, whereas pelagic hauls were much more numerous and covered a much larger area (Fig. 1).

Another peculiarity of our checklist pointed out in the previous section is related to the selectivity of trawls. The number of fish species in the list is similar to, or somewhat higher than, that of invertebrates (Figs 2 and 3), whereas total species richness of invertebrates is much higher than that of fishes, based on data from different sampling gear^90^. This is not unexpected, since fewer fish species can be sampled, for example, by a benthic grab sampler than by a trawl, and *vice versa* for small and burrowing forms. As a result, polychaetes and bivalves often dominate the grab benthos; whereas large gastropods such as the Buccinidae dominate the trawl benthos (Table 7, Figs 2 and 3).

Further detailed analyses of Figs 2 and 3 were omitted, deferring to future analysis by specialists in particular taxa.

### Comparison of basins by species richness

**Figure 4:**
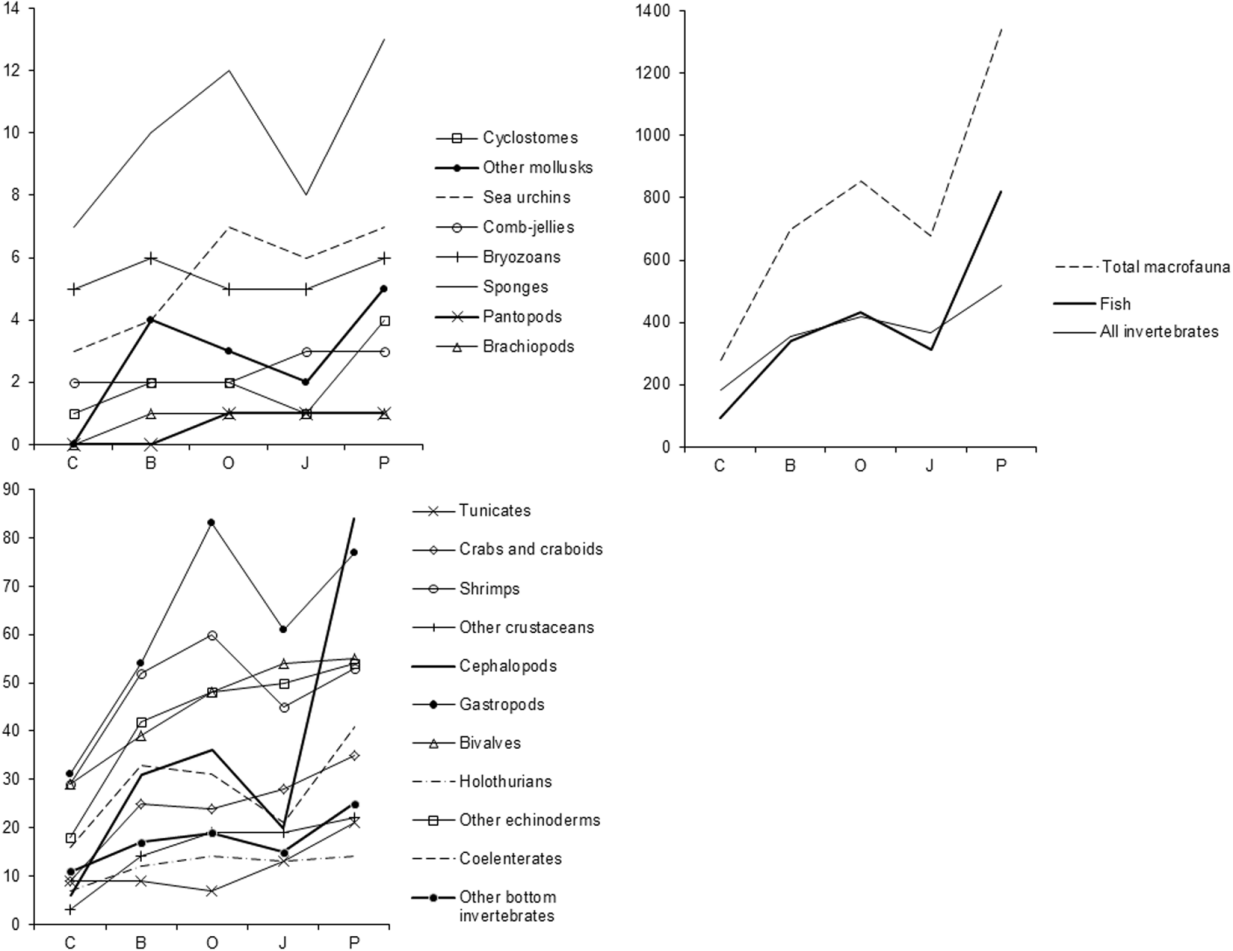
Relationship of species richness by basins. Species richness values differ greatly among different species groups, so the data is displayed among panels at three different scales.

Species richness is expected to increase from the Chukchi Sea to the Sea of Japan and further into the Pacific Ocean, following the Humboldt-Wallace rule^91–98^. That is, proceeding from the poles to the equator, it indeed increases from north to south, following the decrease in latitude and increase in water temperature (Table 7, Fig. 4). However, for all macrofauna and many taxa, this generalization fails for the Sea of Japan, where species richness is significantly lower than expected: for most higher taxa (55%) it is lower than in the Sea of Okhotsk and for some taxa (40%) even lower than in the Bering Sea.

This phenomenon may have several explanations, each not necessarily contradicting the others. First, our data come from only the subarctic northwestern Sea of Japan (Fig. 1): there were no trawl surveys in the eastern and southern parts, where water temperature is higher and species richness is significantly higher. Second, for an extended geological period, the Sea of Japan was a shallow-water isolated basin. At present this basin is deep, with a narrow shelf and low temperature in deep-sea areas, isolated from deep-sea areas of adjacent basins by relatively narrow and shallow straits. Therefore the species richness of deep-sea fauna in the Sea of Japan is lower than in adjacent seas and the Pacific Ocean^1,99^. This is also true for fishes: there are 50 macrourid species known in the Pacific Ocean off Japan, and only one or two in the Sea of Japan; 33 myctophid species on the Pacific Ocean side and two in the Sea of Japan^99^. Third and finally, the area surveyed in the Sea of Japan is much smaller compared to that in the Bering Sea, the Sea of Okhotsk and the Pacific Ocean (Table 1).

The general trend is not observed also in the Sea of Okhotsk for gastropods, sea urchins or sponges, which exhibit higher than expected species richness, and in the Bering Sea for coelenterates, crabs, “other” molluscs and bryozoans. In fact, there is ample published evidence that there is no worldwide common latitudinal trend for most taxa, regions, ocean depths (vertical zones) or geographical scales^94,100–110^. Recent studies have, instead, identified a small number of hot spots of species richness in the ocean^110–112^, interpreted by Mironov^112^ as “centres of redistribution of fauna” (formerly known as centres of faunal origin, centres of accumulation or dispersal), and species richness generally declines with distance from these centres. However, the data in the present paper reveal evidence for an increase in trawl macrofauna species richness in the survey area from north to south (with the few exceptions noted) but this trend is not in conflict either with the Humboldt-Wallace rule, or with the position of the Indo-Malayan centre of faunal redistribution.

### Correlation of species richness with sample size and species-area ratio

The initial data for the analysis of species richness dependent on the sample size were obtained by including into each row of Table 1 the number of species found in a given sample from the 3rd column of Table 3, calculated using the checklist (Supplementary Table), taking into account only trawl survey data indicated by “+”. (Everywhere else in the paper, species richness for any habitat and group of animals is determined by taking into account both “+” (actual data) and “*” (published data), as in the 5th column of Table 3).

**Figure 5:**
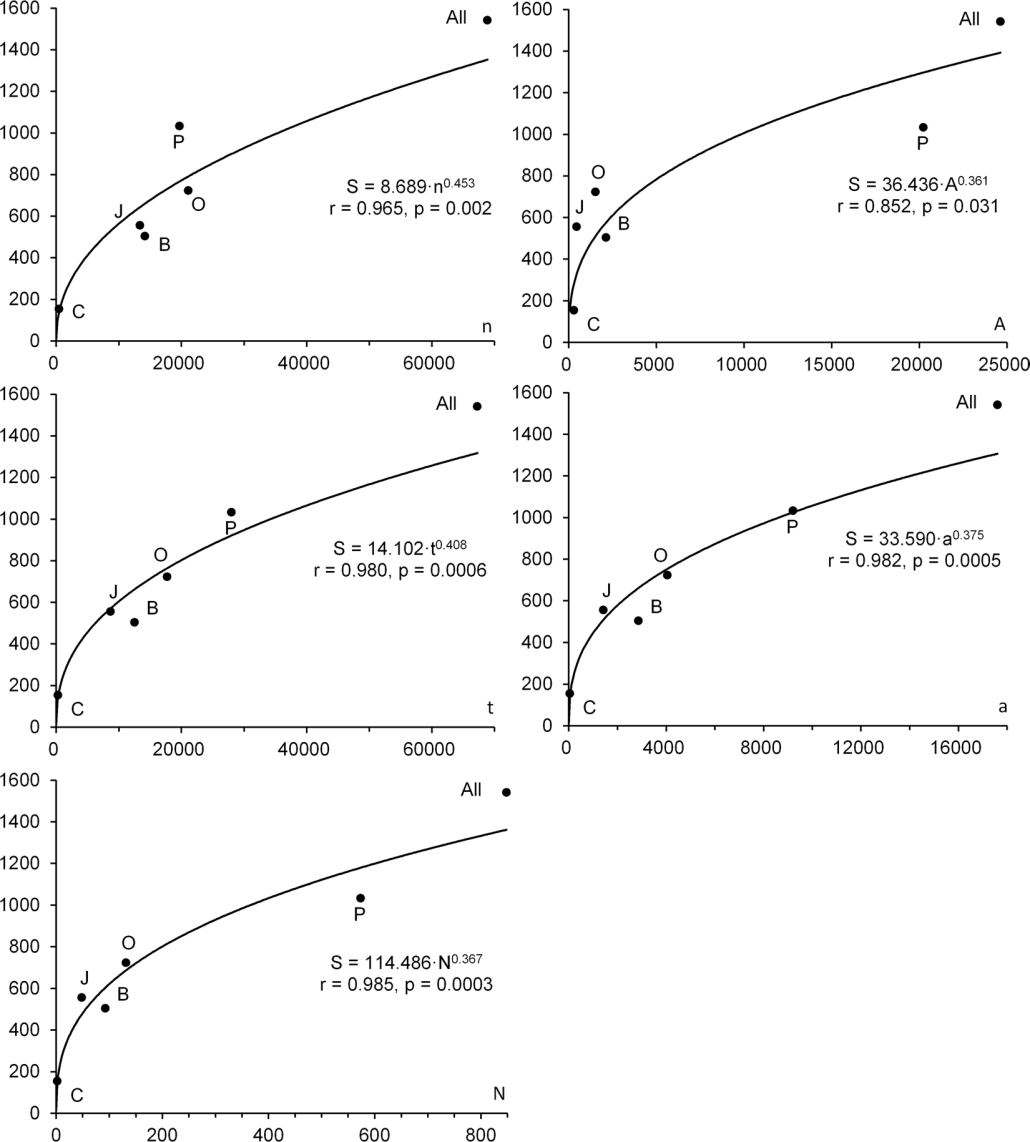
Relationship of species richness S and sample size characteristics: A, survey area; a, total study area; N, number of individuals captured; n, number of trawl hauls; and t, total trawling time. Basins are coded as in legends to Figs 1 and 3 and the text. Pearson’s r correlation coefficient and p-value are given for each equation.

The species richness appears to be positively and statistically significantly related to all five of the sample size characteristics by which it was estimated (Fig. 5). All these relationships are satisfactorily described by multiplicative equations such as y = *a*·x^*b*^. Based on an increase in *r* and a decrease in *p*-values, the relationship with surveyed area was the weakest; somewhat stronger with the number of samples taken; stronger with total survey time; very strong with total sample area; and strongest with the number of caught and identified individuals.

These findings correspond to the earlier hypothesis^107^ that an increase in sample size leads to an increase in species richness estimates associated with (1) comprehensiveness and (2) intensity of data collection.

1. As the area surveyed increases, new types of habitats with their inherent species are included, so species richness grows according to the long-standing and well-known “species-area” law^113,114^.
2. At low levels of species evenness, as with the fauna under consideration here^115^, many species occur rarely or very rarely. Therefore, no matter how large a sample is taken, there is always a chance that one more captured individual will belong to a rare species, still absent from the sample, even if the survey area does not expand and samples are taken in the same places. As a consequence, the more samples taken, the more time spent on sampling, the larger the total sample size (the area and volume surveyed by trawls), the number of individuals in samples used for estimation of species richness and, consequently, the higher the resulting species richness. It is worth noting that the effect of the listed sample characteristics on species richness decreases in reverse order (see Fig. 5). The number of individuals directly affects species richness (the strongest relationship). The influence of the total sample size is weakened due to the unequal number of individuals in each sample. The survey time is not directly proportional to the size of a sample, and moreover, to the number of individuals sampled. Finally, the number of samples (the weakest relationship) is a sample characteristic with the maximum uncertainty, since trawl hauls vary greatly in duration, speed, opening of the trawl mouth and catch size. In particular, this explains the above-mentioned phenomenon: fewer pelagic stations provide better comprehensiveness for pelagic surveys, and more stations are required for bottom surveys, since all other sample size features in the water column are larger than at the seafloor (see Table 1).

**Figure 6:**
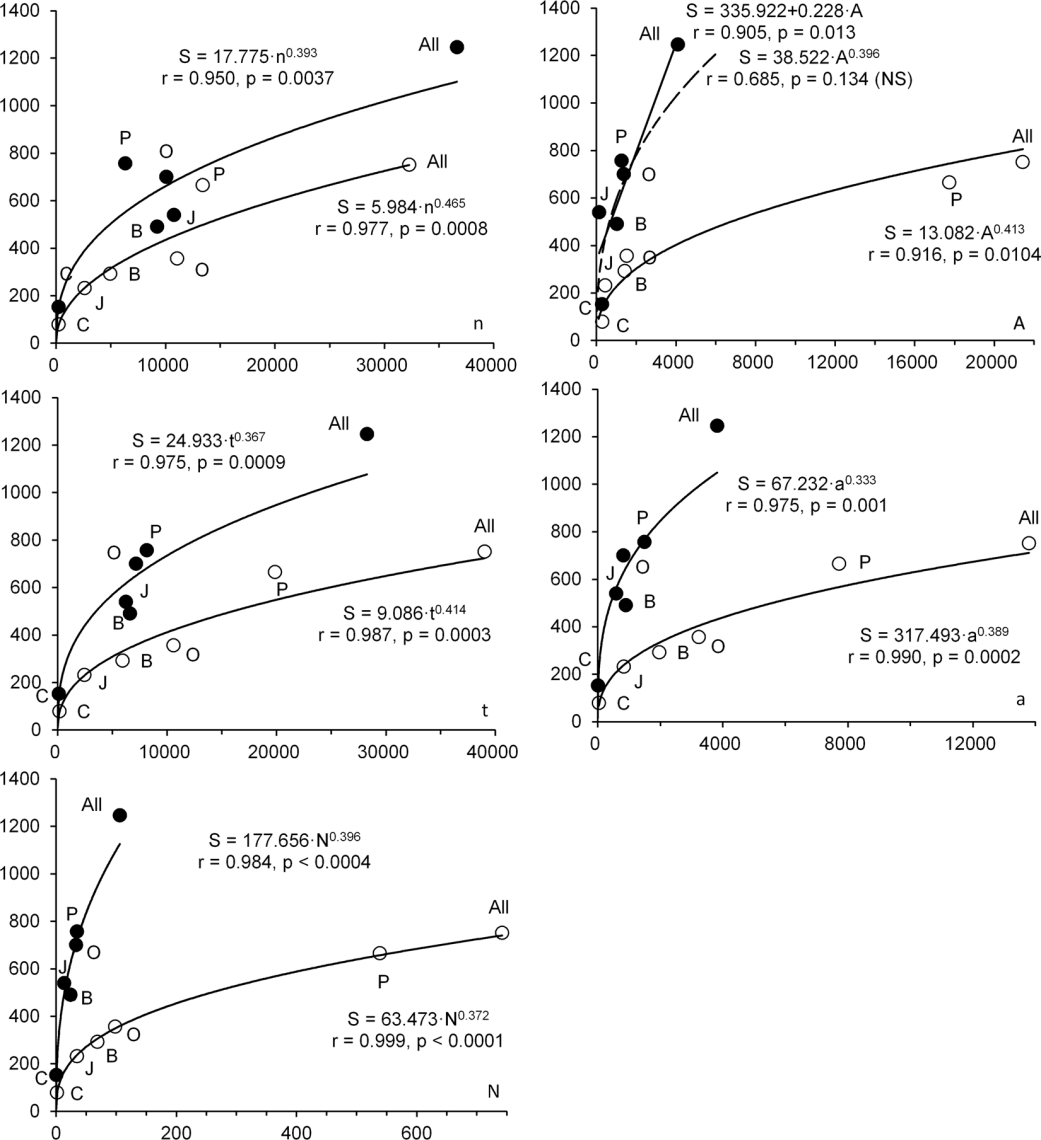
Relationship between species richness in the pelagic (open circles) and benthic (dark circles) zones and sample size characteristics. Lettering as in the Fig. 5. Multiplicative relationships between S and A are statistically insignificant (dashed line at top of figure) and are better described by linear regression for bottom samples.

The same analysis was repeated taking the pelagic and seafloor data separately and similar results were obtained (Fig. 6). It was also found that species richness correlates with all sample size features more strongly in the pelagic zone than on the seabed. In general, there are more species at the seafloor than in the water column. In almost all cases, the relationships among variables are satisfactorily described by the multiplicative model and, in all equations, the value of the slope is close to 0.4. The exception is the “species-area” relationship in the benthic zone, which is better approximated by a simple linear model.

We recalculated five pairs of equations (Fig. 6) in the form of a multiplicative model with degree (slope) *b* the same for every pair “water column/bottom” (since in each pair the differences between these values are statistically insignificant) and factor (intercept) *a* different. On logarithmic scales, the regression lines of each pair were parallel straight lines, the values of *b* for different pairs (the upper half of Table 8) varied from 0.361 to 0.429 and did not significantly differ from 0.4. The values of *a* for each pair differed by a factor of 1.6–3.0.

**Table 8:** Regression coefficients for equation S = a·x^b^, where S – species richness, x – different sample size values from Table 1.

Condition	Independent variable (x)	Slope (*b*)	Intercept (*a*)	
			Pelagic	Benthic
*a* and *b* are estimated, *b* for pelagic zone and bottom is the same	Number of samples, n	0.429	8.134	12.910
	Study area, A	0.408	13.632	35.582
	Total trawling time, t	0.392	11.003	20.321
	Total area covered by trawls, a	0.361	21.453	56.352
	Number of captured individuals, N	0.381	60.959	185.293
only *a* is estimated, *b* - constant	Number of samples, n	0.400	11.538	17.166
	Study area, A	0.400	14.098	40.843
	Total trawling time, t	0.400	10.830	19.215
	Total area covered by trawls, a	0.400	16.510	42.940
	Number of captured individuals, N	0.400	53.997	180.139

A series of calculations was also made (the lower half of Table 8) in which *b* in all equations was a constant of 0.4 (i.e. all regression lines are parallel on logarithmic scales), and only the value of *a* was estimated (distance along the ordinate between parallel lines). At the same time, in each pair “water column/bottom”, the values of *a* differed by a factor of 1.5–3.3. The results of these calculations show that estimates for species richness will increase with increasing extent and intensity of surveys, but with equal effort will yield 2–3 times more species on the seafloor than in the water column.

### Comparison of basins by species composition

**Figure 7:**
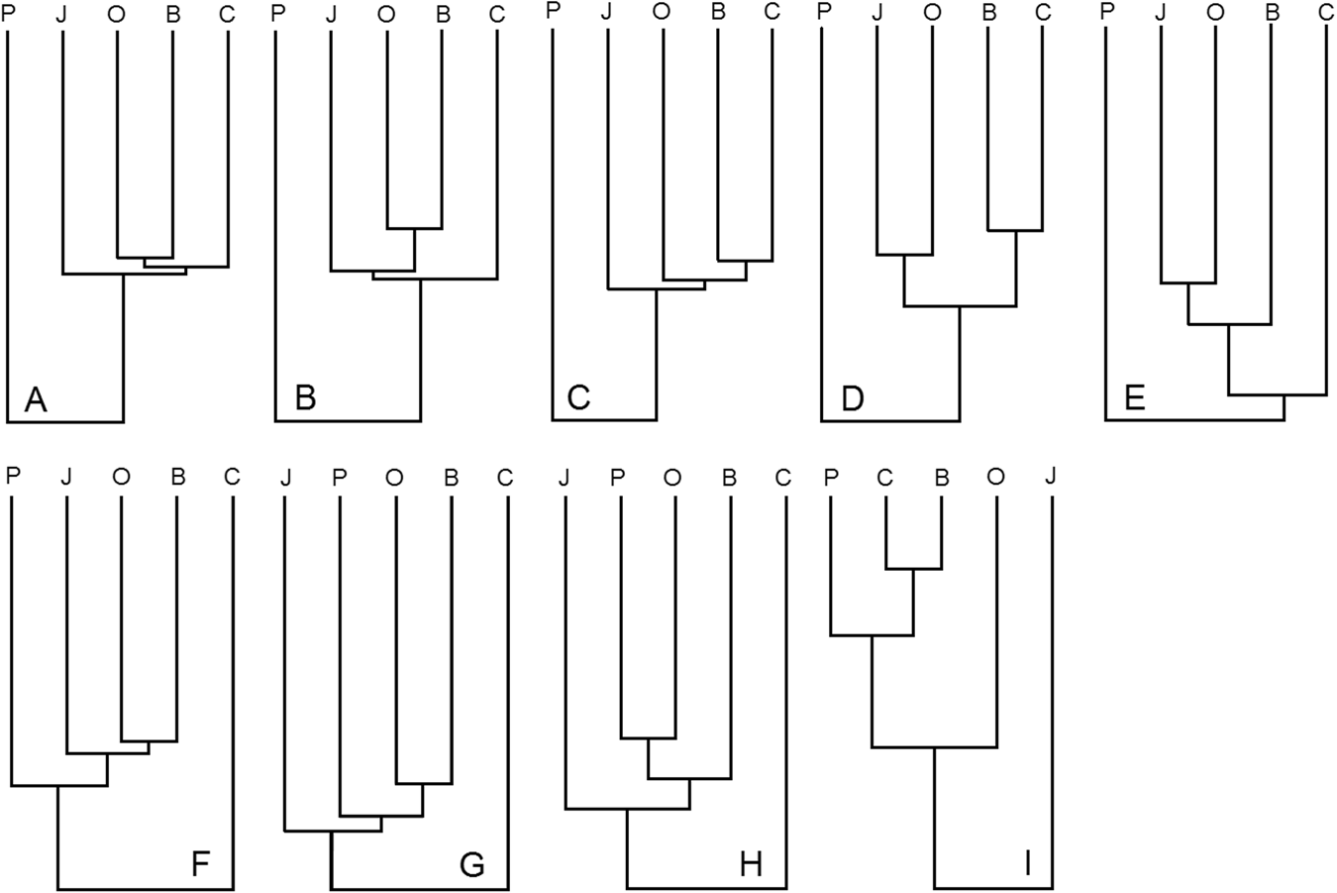
Nine variants (A–I) of clustering of basins based on species composition according to different measures and the algorithms listed in Table 9.

Cluster analyses of the species lists using various similarity measures and algorithms for constructing dendrograms are summarized in Table 9 and Fig. 7. The results are inconclusive: first, the SL and UPA algorithms often yielded different results; and second, measures of the first type (Nos 1–5 in Table 9) take into account both presence and absence of a species, so the Pacific Ocean, with the longest species list, differs most strongly from all the other seas investigated (Fig. 7A–E). In the most common scenarios of clustering (A and B), the Bering and Okhotsk seas are most similar. The difference between A and B is in how similar to them is the Chukchi Sea (A) or the Sea of Japan (B). Measures of the second type (Nos 6–13 in Table 9) are characterized by separation not of the Pacific Ocean but of the Chukchi Sea (Fig. 7F–H) or the Sea of Japan (I). In the most common cases (F and G), the Bering and Okhotsk seas are also the closest in species composition. The difference between F and G is in how similar to this pair is the Sea of Japan or the Pacific Ocean.

**Table 9:** Classification of nine outputs (A–I) for comparison of trawl macrofauna composition among basins according to different approaches: 13 measures of similarity and 2 clustering algorithms (SL – single linkage, UPA – unweighted pair-group average). Resulting dendrograms (A-I) are shown in Fig. 7.

Type	Measure of similarity	Reference	No	Clustering algorithm	
	Formula			SL	UPA
Coefficients treating *a* and *d* symmetrically	$\frac{a+d}{a+b+c+d}$	^120^	1	A	B
	$\frac{a+d}{a+2b+2c+d}$	^121^	2	A	B
	$\frac{2a+2d}{2a+b+c+2d}$	^122^	3	A	B
	$\frac{1}{4}\cdot (\frac{a}{a+b}+\frac{a}{a+c}+\frac{d}{b+d}+\frac{d}{c+d})$	^122^	4	C	D
	$\frac{a}{\sqrt{(a+b)(a+c)}}\cdot \frac{d}{\sqrt{(b+d)(c+d)}}$	^122^	5	E	E
Coefficients ignoring the value of *d*	$\frac{a}{a+b+c}$	^123^	6	F	G
	$\frac{2a}{2a+b+c}$	^124– 126^	7	F	G
	$\frac{3a}{3a+b+c}$	^86^	8	F	G
	$\frac{a}{a+2b+2c}$	^122^	9	F	G
	$\frac{1}{2}\cdot (\frac{a}{a+b}+\frac{a}{a+c})$	^127^	10	F	G
	$\frac{a}{\sqrt{(a+b)(a+c)}}$	^128, 129^	11	F	G
	$\frac{a}{b+c}$	^127^	12	H	H
	$\frac{a}{{\rm{\min }}(a+b,\,a+c)}$	^130^	13	I	I

Results based on binary similarity coefficients No. 1–3 were also obtained using five distances (dissimilarity coefficients) commonly used for abundance data – Euclidean, Gower, Hemming, Manhattan, and Jukes-Kantor. Measure 4 produced the same results as 1-Pearson’s and 1-Spearman’s correlations. Measures 6–11 yielded the same results as five distances – Chord, Bray-Curtis, Cosine, Morisita, and Horn. Ward’s clustering algorithm was also applied but the output was similar to UPA and so is omitted here.

As a result, taking into account rare cases (C–E, H, I), we obtained nine different scenarios. We tried to reduce uncertainty in the results by analysing separately bottom and pelagic fauna or exclusively the fish fauna using the same methods, but the same scenarios plus a few more variants were obtained. Theoretically, any given method (the combination of a distance measure and a clustering algorithm) is no better than any other: they are not able to check statistical hypotheses about the adequacy of resulting classifications. Different methods of clustering give the same (or very similar) results where analyzed data sets are clearly divided into natural groups. The less clear the differences between the groups, the larger the number of specific clustering results that need to be checked-up in order to determine in them meaningful and predictive patterns^116^.

It is suggested that clustering methods that take into account species occurrence or abundance (e.g.^117^), might have yielded less ambiguous results, but this is a task for a separate study, the initial data of which go beyond the simple species list presented herein. We can only use as a basis the most frequent variants A, B, F and G, since the differences between them are relatively small and easily understood (see above)). Beyond these, the results should be checked using other non-hierarchical methods of multivariate analyses (cf. the recommendation of Kafanov *et al*.^116^).

**Figure 8:**
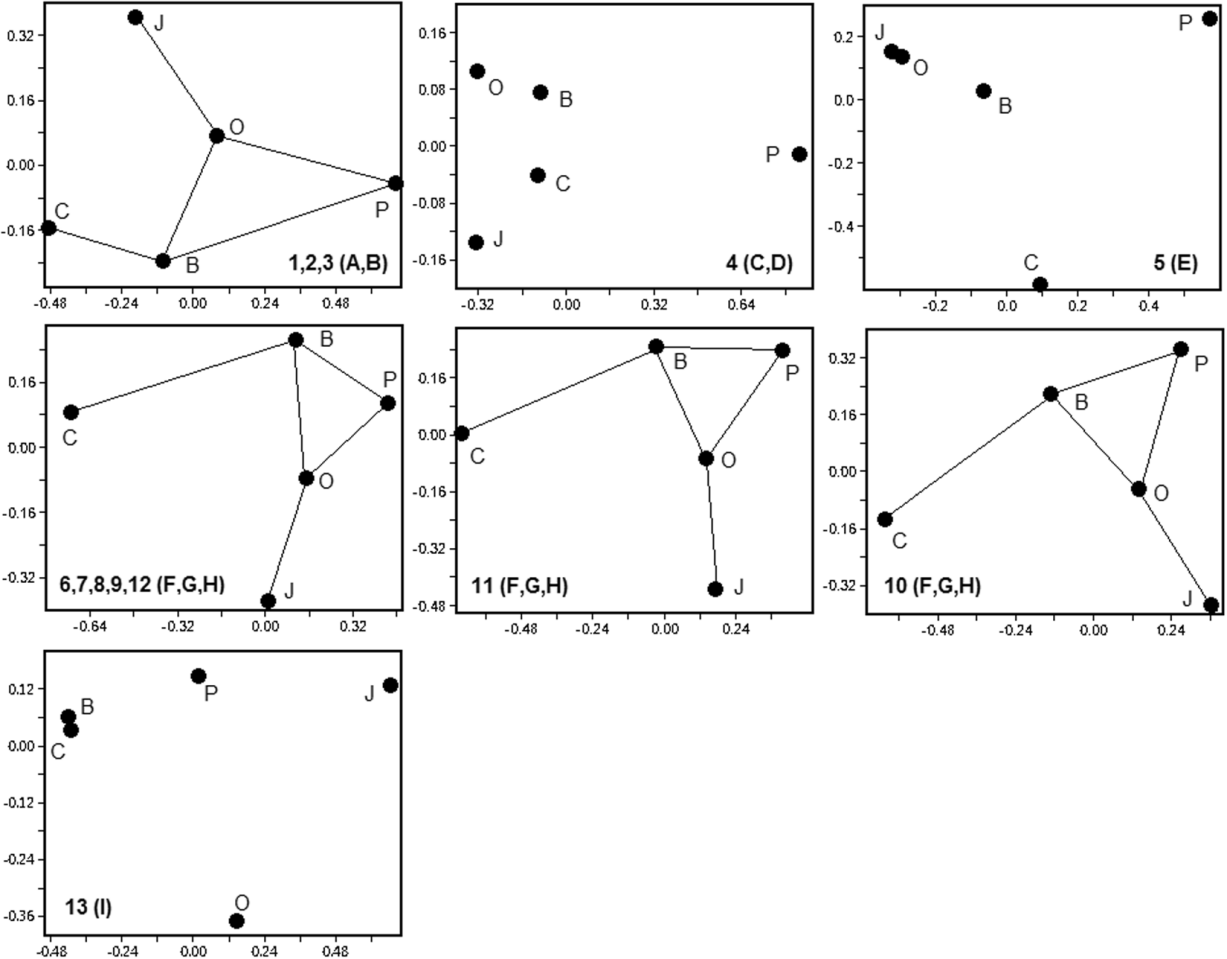
Seven versions of non-metric multidimensional scaling of basins by species composition. Measures of similarity from Table 9 are shown at the base of each graph; letters in parentheses indicate corresponding clustering variants as in Table 9 and Fig. 7. The closest points are connected by lines on similar graphs.

For check-up we used the non-metric multidimensional scaling (MDS): the algorithm based on the approach developed by Taguchi and Oono^118^; and the principal coordinates analysis (PCO) also known as the metric multidimensional scaling (MMS), the algorithm from Davis^119^. The results from using these methods coincided almost completely, so here, only the results from MDS are shown (Fig. 8) and discussed.

Measures of the first group (Nos 1–3) yielded results similar to those measures 6–9 and 12, if the y-axis is reversed. Along this axis, B, O and J are separated by similar distances. The only noticeable difference is that, for measures 1–3), this first group is located closer to C, whereas (for measures 6–9, 12) it is shifted along the x-axis towards P. Measure 11 yielded results similar to those of measures 6–9 and 12, but rotated counter-clockwise. Applying measure 10 gave a similar result, the only difference being a slightly larger angle of rotation. The results of measures 4, 5 and 13 differed from all the others.

**Figure 9:**
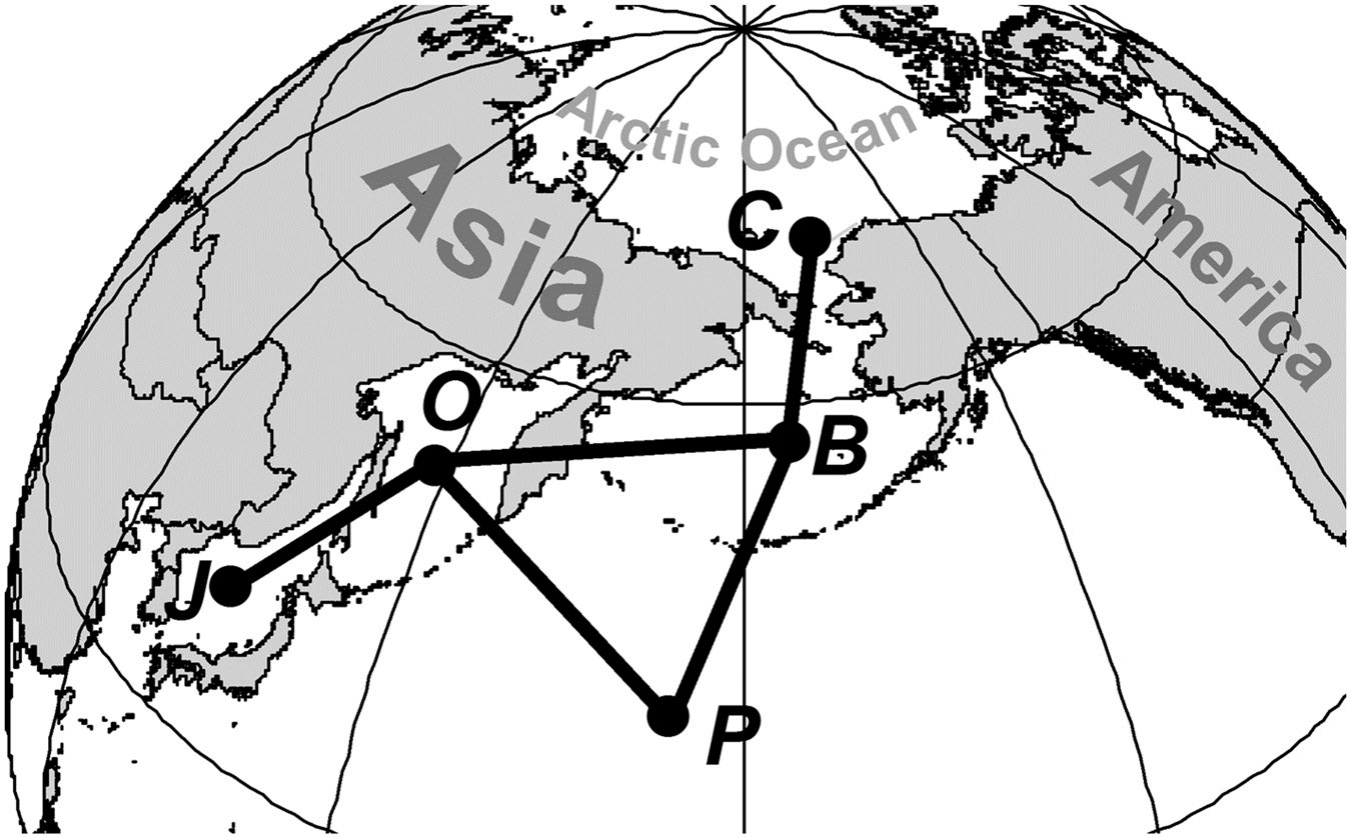
Projection of the circuit from Fig. 8 to the map of the surveyed water area. Basins are coded as in legends to Figs 1 and 3 and the text.

Therefore, in ten out of thirteen measures, the points B, O and P form an almost equilateral triangle, with J and C outside it and farther from P; C closer to B; and J closer to O. This combination in general corresponds to the most frequent dendrograms (Fig. 7A,B,F and G), and also to the remoteness of these basins from each other (Fig. 9), the water exchange between them, animal migration possibilities, and faunal mixing, etc.

## Conclusions

The present paper provides examples of the analysis of information present in the checklist compiled, revealing the following points of interest:

1. Trawls catch approximately 23% of all species of macrofauna (“the trawl macrofauna” in the presented checklist). These include all Cyclostomata species, 65% of fish species and not more than 11% of invertebrate species from the examined area.
2. These percentages vary among basins and taxa. They are positively related to sample size (i.e. effort spent to examine a particular area) and negatively related to catching efficiency of a given trawl for a particular taxon.
3. Despite the enormous amount of material collected, the compiled list of 1,541 species is not complete. It will grow both with expansion of the study area and with continuing research in the area already examined, owing to future addition of rare species and/or species with low catching efficiency.
4. Such an increase in the number of species will be largely due to the near-bottom species, since their number is 2–3 times higher than that of pelagic species, and the pelagic zone is better studied than the benthic. Among all basins, the greatest increase in the species number can be expected in the Sea of Japan, since the trawl macrofauna of that region in Russia is inadequately studied.
5. Fishes and cephalopods dominated the pelagic trawl catches, whereas benthic trawls were dominated by invertebrates, most of which do not occur in the pelagic zone.
6. The number of species in trawl catches increased from north to south, in line with the Humboldt-Wallace rule and location of the Pacific Ocean center of species redistribution in the Indo-Malayan archipelago area.
7. Based on the trawl macrofauna composition, the Bering Sea and the Sea of Okhotsk are most similar to the Pacific Ocean; the Chukchi Sea is similar to the Bering Sea; and the northwestern Sea of Japan is similar to the Sea of Okhotsk.

In the future, more valuable information can be obtained from the checklist presented herein using other methods of data processing and/or addition of data (such as abundance, occurrence and catches). Comparisons with similar lists from other areas or with lists from the same area obtained using different techniques also may be of interest. The list published here should be of interest to ichthyologists, hydrobiologists, ecologists, biogeographers, conservation biologists and fishery managers, as well as teachers and students of relevant specialties.

## Electronic supplementary material

Supplementary Dataset 1
