# Supplementary material for: Species richness and taxonomic composition of trawl macrofauna of the North Pacific and its adjacent seas
Source: Sci Rep. 2018 Nov 9;8:16604. doi: 10.1038/s41598-018-34819-4 (PMC6226505; doi:10.1038/s41598-018-34819-4)
Supplement: Supplementary file 1 — Supplementary Dataset 1 [file 41598_2018_34819_MOESM1_ESM.docx]

**Species richness and taxonomic composition of trawl macrofauna of the North Pacific and its adjacent seas**

*Igor V. Volvenko*^1^, Alexei M. Orlov^2,3,4,5,6^, Andrey V.* *Gebruk^7^, Oleg N. Katugin^1^, Georgy M. Vinogradov^7^, Olga A. Maznikova^2^*

^1^Pacific Research Fisheries Center (TINRO-Center), Vladivostok, 690091 Russia; ^2^Russian Federal Research Institute of Fisheries and Oceanography (VNIRO), Moscow, 107140 Russia; ^3^A.N. Severtsov Institute of Ecology and Evolution, Russian Academy of Sciences (IPEE), Moscow, 119071 Russia; ^4^Dagestan State University (DSU), Makhachkala, 367000 Russia; ^5^Tomsk State University (TSU), Tomsk, 634050 Russia; ^6^Caspian Institute of Biological Resources, Dagestan Scientific Center, Russian Academy of Sciences (CIBR DSC RAS), Makhachkala, 367023 Russia; ^7^P.P. Shirshov Institute of Oceanology, Russian Academy of Sciences (IO RAS), Moscow, 117997 Russia

*Correspondence**:** Igor V. Volvenko, TINRO-Center, 4 Shevchenko Ave., Vladivostok, 690091, Russia, Tel. +7 423 2400451, E-mail [oknevlov@gmail.com](mailto:volvenko@tinro.ru)

**Supplementary material**

**Supplementary Table** Checklist of the trawl macrofauna from the study area (see Fig. 1) based on the dataset of the Pacific Research Fisheries Center (TINRO-Center, Vladivostok). Gear: M – midwater trawl, B – bottom trawl. Basins: C – Chukchi Sea, B – Bering Sea, O – Sea of Okhotsk, J – Sea of Japan, P – Pacific Ocean, “–“ – absent, “+” present, * – known from the basin based on publications, but not present in our samples.

| Species, genus or family | Taxon | Gear | Region | | | | |
| --- | --- | --- | --- | --- | --- | --- | --- |
|  |  |  | C | B | O | J | P |
| *Abralia andamanica* | 7 | M | – | – | – | – | + |
| *Abralia similis* | 7 | M | – | – | – | – | + |
| *Abraliopsis felis* | 7 | M | – | – | – | – | + |
| *Abraliopsis pacificus* | 7 | M | – | – | – | – | + |
| *Acanthogobius flavimanus* | 1 | B | – | – | – | + | – |
| *Acantholumpenus mackayi* | 1 | MB | – | + | + | + | * |
| *Acanthopsetta nadeshnyi* | 1 | MB | – | + | + | + | + |
| *Achaetobonellia maculata* | 20 | B | – | – | – | + | * |
| *Acila insignis* | 9 | B | – | – | * | + | * |
| *Acipenser medirostris* | 1 | B | – | – | – | + | + |
| *Actinauge verrillii* | 14 | B | – | * | + | – | * |
| *Actinia sp.* | 14 | B | * | * | + | + | * |
| *Adelosebastes latens* | 1 | B | – | – | – | – | + |
| *Aequorea coerulescens* | 14 | B | – | – | – | + | * |
| *Aforia circinata* | 8 | B | – | + | * | * | * |
| *Aglantha digitale* | 14 | MB | * | + | * | * | + |
| *Agonomalus jordani* | 1 | MB | – | + | + | + | + |
| *Agonomalus proboscidalis* | 1 | MB | – | – | + | + | + |
| *Agonopsis vulsa* | 1 | B | – | + | – | – | – |
| *Ahliesaurus brevis* | 1 | M | – | – | – | – | + |
| *Albatrossia pectoralis* | 1 | MB | – | + | + | – | + |
| *Alcichthys elongatus* | 1 | MB | – | – | + | + | + |
| *Alcyonidium gelatinosum* | 16 | B | + | * | * | * | * |
| *Alcyonidium vermiculare* | 16 | B | + | * | – | * | – |
| *Aldrovandia phalacra* | 1 | B | – | – | – | – | + |
| *Alectrias alectrolophus* | 1 | B | * | * | + | + | * |
| *Alectrias cirratus* | 1 | B | – | – | – | + | – |
| *Alectrias gallinus* | 1 | B | – | – | * | + | * |
| *Alepisaurus ferox* | 1 | MB | – | + | + | – | + |
| *Alepocephalus tenebrosus* | 1 | B | – | * | – | – | + |
| *Alepocephalus umbriceps* | 1 | B | – | – | + | – | + |
| *Alitta virens* | 20 | B | – | – | + | – | * |
| *Allocareproctus jordani* | 1 | B | – | * | + | – | + |
| *Allocyttus folletti* | 1 | B | – | + | – | – | + |
| *Alopias pelagicus* | 1 | M | – | – | – | – | + |
| *Alopias vulpinus* | 1 | M | – | – | – | – | + |
| *Alosa sapidissima* | 1 | B | – | + | – | – | + |
| *Amathillopsis sp.* | 6 | MB | – | – | + | – | + |
| *Amblyraja badia* | 1 | B | – | * | + | – | + |
| *Ammodytes hexapterus* | 1 | MB | + | + | + | + | + |
| *Ammodytes personatus* | 1 | B | – | + | + | * | + |
| *Amphiodia fissa* | 13 | B | – | – | + | + | + |
| *Amphitretus pelagicus* | 7 | M | – | – | – | – | + |
| *Anadara broughtonii* | 9 | B | – | – | – | + | * |
| *Anarhichas orientalis* | 1 | MB | + | + | + | + | + |
| *Anarhichthys ocellatus* | 1 | MB | – | + | – | – | + |
| *Ancistrocheirus lesueurii* | 7 | M | – | – | – | – | + |
| *Ancistrolepis grammatus* | 8 | B | – | – | + | – | * |
| *Ancistrolepis kawamurai* | 8 | B | – | – | + | – | * |
| *Anguilla sp.* | 1 | B | – | – | + | – | – |
| *Anisarchus macrops* | 1 | B | – | – | – | + | * |
| *Anisarchus medius* | 1 | MB | + | + | + | + | * |
| *Anoplagonus occidentalis* | 1 | MB | – | – | * | + | – |
| *Anoplogaster cornuta* | 1 | MB | – | + | + | – | + |
| *Anoplopoma fimbria* | 1 | MB | – | + | + | – | + |
| *Anotopterus nikparini* | 1 | MB | – | + | + | – | + |
| *Anthias sp.* | 1 | B | – | + | – | – | + |
| *Antigonia capros* | 1 | M | – | – | – | – | + |
| *Antigonia eos* | 1 | B | – | – | – | – | + |
| *Antimora microlepis* | 1 | MB | – | + | + | – | + |
| *Aphanopus arigato* | 1 | MB | – | – | – | – | + |
| *Aphelasterias japonica* | 13 | B | – | – | – | + | * |
| *Aphrodita aculeata* | 20 | B | – | * | + | – | * |
| *Aphrodita australis* | 20 | B | + | – | + | + | + |
| *Aphrodita californica* | 20 | B | – | – | + | – | * |
| *Aphrodita negligens* | 20 | B | – | * | + | – | * |
| *Aplidium sp.* | 3 | B | * | * | * | + | * |
| *Aplysia sp.* | 8 | B | – | – | – | + | * |
| *Apostichopus japonicus* | 12 | B | – | – | – | + | * |
| *Apristurus brunneus* | 1 | B | – | – | – | – | + |
| *Apristurus fedorovi* | 1 | MB | – | – | – | – | + |
| *Apristurus macrorhynchus* | 1 | B | – | – | – | – | + |
| *Apristurus platyrhynchus* | 1 | MB | – | – | – | – | + |
| *Aptocyclus ventricosus* | 1 | MB | + | + | + | + | + |
| *Arca boucardi* | 9 | B | – | – | * | + | * |
| *Architeuthis dux* | 7 | M | – | – | – | – | + |
| *Arctoscopus japonicus* | 1 | MB | – | – | + | + | + |
| *Arctozenus risso* | 1 | MB | – | + | + | – | + |
| *Arcturus hastiger* | 6 | B | – | – | + | + | – |
| *Arcturus setosus* | 6 | B | – | – | * | – | + |
| *Argentina sialis* | 1 | B | – | – | – | – | + |
| *Argis crassa* | 5 | MB | * | + | + | + | * |
| *Argis dentata* | 5 | MB | + | + | + | + | – |
| *Argis hozawai* | 5 | B | – | – | * | + | – |
| *Argis lar* | 5 | MB | + | + | + | + | + |
| *Argis ochotensis* | 5 | B | – | + | + | + | – |
| *Argis ovifer* | 5 | MB | – | + | + | + | + |
| *Argis toyamaensis* | 5 | B | – | – | – | + | – |
| *Argonauta argo* | 7 | M | – | – | – | * | + |
| *Argonauta nouryi* | 7 | M | – | – | – | – | + |
| *Argyripnus ephippiatus* | 1 | M | – | – | – | – | + |
| *Argyrocottus zanderi* | 1 | B | – | – | * | + | * |
| *Argyropelecus aculeatus* | 1 | MB | – | – | – | – | + |
| *Argyropelecus affinis* | 1 | M | – | – | – | – | + |
| *Argyropelecus hemigymnus* | 1 | M | – | – | – | – | + |
| *Argyropelecus lychnus* | 1 | M | – | – | – | – | + |
| *Argyropelecus sladeni* | 1 | MB | – | + | – | – | + |
| *Argyrosomus japonicus* | 1 | M | – | – | – | – | + |
| *Ariomma luridum* | 1 | B | – | – | – | – | + |
| *Aristostomias scintillans* | 1 | M | – | – | + | – | + |
| *Arothron firmamentum* | 1 | M | – | – | – | – | + |
| *Artediellichthys nigripinnis* | 1 | B | – | + | + | – | + |
| *Artediellina antilope* | 1 | B | – | – | + | – | – |
| *Artediellus aporosus* | 1 | MB | – | – | + | + | – |
| *Artediellus atlanticus* | 1 | B | + | – | – | – | – |
| *Artediellus camchaticus* | 1 | B | – | + | + | – | + |
| *Artediellus dydymovi* | 1 | MB | – | + | + | + | + |
| *Artediellus gomojunovi* | 1 | B | * | + | – | – | * |
| *Artediellus miacanthus* | 1 | B | – | + | + | – | + |
| *Artediellus minor* | 1 | B | – | – | – | + | – |
| *Artediellus ochotensis* | 1 | MB | * | + | + | + | – |
| *Artediellus pacificus* | 1 | B | * | + | – | – | + |
| *Artediellus scaber* | 1 | MB | + | + | – | – | – |
| *Artediellus schmidti* | 1 | MB | – | – | + | + | + |
| *Ascidia sp.* | 3 | B | * | + | * | + | * |
| *Askoldia variegata* | 1 | B | – | + | + | + | + |
| *Aspidophoroides monopterygius* | 1 | MB | + | + | + | + | + |
| *Aspidophoroides olrikii* | 1 | MB | + | + | – | – | – |
| *Assurger anzac* | 1 | M | – | – | – | – | + |
| *Astarte alaskensis* | 9 | B | * | * | * | + | * |
| *Astarte borealis* | 9 | B | + | * | – | + | * |
| *Astarte elliptica* | 9 | B | – | * | * | – | + |
| *Asterias amurensis* | 13 | B | * | + | + | + | + |
| *Asterias argonauta* | 13 | B | – | – | – | + | – |
| *Asterias rathbuni* | 13 | B | * | * | + | – | – |
| *Asterias rollestoni* | 13 | B | – | – | + | + | * |
| *Asteronyx loveni* | 13 | B | – | + | + | * | + |
| *Astronesthes fedorovi* | 1 | M | – | – | – | – | + |
| *Astronesthes ijimai* | 1 | M | – | – | – | – | + |
| *Astronesthes indicus* | 1 | MB | – | – | – | – | + |
| *Astronesthes indopacificus* | 1 | M | – | – | – | – | + |
| *Astronesthes lucifer* | 1 | MB | – | – | – | – | + |
| *Astronesthes nigroides* | 1 | M | – | – | – | – | + |
| *Atheresthes evermanni* | 1 | MB | – | + | + | + | + |
| *Atheresthes stomias* | 1 | MB | – | + | + | – | + |
| *Atolla wyvillei* | 14 | MB | – | + | + | – | + |
| *Aulacofusus sp.* | 8 | B | * | * | + | – | + |
| *Aurelia aurita* | 14 | MB | * | + | + | + | + |
| *Aurelia labiata* | 14 | M | – | + | + | + | + |
| *Aurelia limbata* | 14 | MB | + | + | + | + | + |
| *Auxis rochei* | 1 | M | – | – | – | * | + |
| *Auxis thazard* | 1 | M | – | – | – | + | – |
| *Avocettina infans* | 1 | MB | – | + | + | – | + |
| *Bajacalifornia burragei* | 1 | B | – | – | – | – | + |
| *Bajacalifornia megalops* | 1 | MB | – | – | + | – | + |
| *Balanus rostratus* | 6 | B | * | * | * | + | * |
| *Balistes sp.* | 1 | M | – | – | – | – | + |
| *Barbourisia rufa* | 1 | MB | – | – | – | – | + |
| *Bassozetus zenkevitchi* | 1 | M | – | – | + | – | + |
| *Bathophilus sp.* | 1 | M | – | – | – | – | + |
| *Bathyagonus alascanus* | 1 | B | – | + | – | – | – |
| *Bathyagonus infraspinatus* | 1 | B | – | + | – | – | * |
| *Bathyagonus nigripinnis* | 1 | B | – | + | – | – | + |
| *Bathyagonus pentacanthus* | 1 | B | – | – | – | – | + |
| *Bathycongrus aequoreus* | 1 | B | – | – | – | – | + |
| *Bathygadus antrodes* | 1 | B | – | – | – | – | + |
| *Bathylagus pacificus* | 1 | MB | – | + | + | – | + |
| *Bathylychnops exilis* | 1 | M | – | – | – | – | + |
| *Bathymaster derjugini* | 1 | B | – | * | + | + | – |
| *Bathymaster signatus* | 1 | MB | * | + | + | – | + |
| *Bathyplotes sp.* | 12 | B | – | * | * | – | + |
| *Bathypolypus arcticus* | 7 | B | + | – | – | – | – |
| *Bathypterois sp.* | 1 | B | – | – | – | – | + |
| *Bathyraja abyssicola* | 1 | B | – | + | + | – | + |
| *Bathyraja aleutica* | 1 | MB | – | + | + | – | + |
| *Bathyraja andriashevi* | 1 | B | – | – | + | – | + |
| *Bathyraja bergi* | 1 | B | – | – | + | + | + |
| *Bathyraja diplotaenia* | 1 | B | – | – | * | – | + |
| *Bathyraja fedorovi* | 1 | B | – | – | + | – | + |
| *Bathyraja interrupta* | 1 | B | – | + | – | – | + |
| *Bathyraja isotrachys* | 1 | MB | – | + | + | – | + |
| *Bathyraja maculata* | 1 | MB | – | + | + | – | + |
| *Bathyraja matsubarai* | 1 | B | – | + | + | + | + |
| *Bathyraja minispinosa* | 1 | B | – | + | + | – | + |
| *Bathyraja parmifera* | 1 | MB | – | + | + | + | + |
| *Bathyraja spinosissima* | 1 | B | – | – | + | – | * |
| *Bathyraja trachura* | 1 | B | – | + | + | – | + |
| *Bathyraja tzinovskii* | 1 | MB | – | – | + | – | + |
| *Bathyraja violacea* | 1 | MB | – | + | + | + | + |
| *Bathysphyraenops simplex* | 1 | M | – | – | – | – | + |
| *Bembradium roseum* | 1 | B | – | – | – | – | + |
| *Bembrops filiferus* | 1 | B | – | – | – | – | + |
| *Benthalbella dentata* | 1 | MB | – | + | + | – | + |
| *Benthalbella infans* | 1 | M | – | – | – | – | + |
| *Benthalbella linguidens* | 1 | M | – | – | – | – | + |
| *Bentheogennema borealis* | 5 | B | – | * | + | – | + |
| *Benthoctopus leioderma* | 7 | B | – | * | + | – | + |
| *Benthoctopus profundorum* | 7 | B | – | – | + | + | * |
| *Benthoctopus sibiricus* | 7 | B | + | – | * | – | * |
| *Benthodesmus elongatus* | 1 | MB | – | – | – | – | + |
| *Benthodesmus pacificus* | 1 | M | – | – | – | – | + |
| *Benthodesmus tenuis* | 1 | MB | – | – | – | – | + |
| *Benthosema suborbitale* | 1 | M | – | – | – | – | + |
| *Beringius behringii* | 8 | B | + | + | + | – | * |
| *Beringius frieley* | 8 | B | – | + | – | – | * |
| *Beringius kennicottii* | 8 | B | * | + | – | – | * |
| *Beringius stimpsoni* | 8 | B | * | + | + | + | – |
| *Bero elegans* | 1 | B | – | – | * | + | + |
| *Beroe cucumis* | 15 | M | * | * | * | * | + |
| *Berryteuthis magister* | 7 | MB | * | + | + | + | + |
| *Bertella idiomorpha* | 1 | M | – | – | – | – | + |
| *Beryx decadactylus* | 1 | B | – | – | – | – | + |
| *Beryx splendens* | 1 | MB | – | – | – | – | + |
| *Bilabria ornata* | 1 | B | – | – | * | + | – |
| *Birulia sachalinensis* | 5 | B | – | – | + | * | – |
| *Bispira sp.* | 20 | B | * | * | – | – | + |
| Blenniidae gen. sp. | 1 | MB | + | + | + | + | – |
| *Blepsias bilobus* | 1 | MB | + | + | + | + | + |
| *Blepsias cirrhosus* | 1 | MB | – | + | + | + | + |
| *Bolinichthys photothorax* | 1 | M | – | – | – | – | + |
| *Bolitaena pygmaea* | 7 | MB | – | + | + | – | + |
| *Boltenia echinata* | 3 | B | + | – | + | – | + |
| *Boltenia ovifera* | 3 | B | + | + | + | – | + |
| *Boreogadus saida* | 1 | MB | + | + | – | – | – |
| *Boreoscala greenlandica* | 8 | B | * | * | – | + | * |
| *Boreoteuthis borealis* | 7 | MB | + | + | + | + | + |
| *Boreotrophon candelabrum* | 8 | B | – | – | – | + | * |
| *Bothragonus occidentalis* | 1 | B | – | – | * | + | * |
| *Bothrocara brunneum* | 1 | MB | – | + | + | – | + |
| *Bothrocara hollandi* | 1 | MB | – | + | + | + | + |
| *Bothrocara molle* | 1 | M | – | + | – | – | + |
| *Bothrocara soldatovi* | 1 | MB | – | + | + | + | + |
| *Bothrocarina microcephala* | 1 | MB | – | – | + | – | + |
| *Bothrocarina nigrocaudata* | 1 | MB | – | – | + | – | + |
| *Bougainvillia sp.* | 14 | MB | * | + | – | + | * |
| *Brachioteuthis riisei* | 7 | MB | – | – | – | – | + |
| *Brachyopsis segaliensis* | 1 | B | – | – | + | + | * |
| *Brama japonica* | 1 | MB | – | + | + | + | + |
| *Bregmaceros japonicus* | 1 | M | – | – | – | – | + |
| *Bregmaceros mcclellandi* | 1 | M | – | – | – | – | + |
| *Brephostoma carpenteri* | 1 | M | – | – | – | – | + |
| *Brisaster latifrons* | 13 | B | – | + | + | + | * |
| *Bryozoichthys lysimus* | 1 | B | – | + | + | + | + |
| *Buccinum acutispiratum* | 8 | B | – | – | + | * | – |
| *Buccinum angulosum* | 8 | B | + | + | + | – | * |
| *Buccinum argillaceum* | 8 | B | – | – | + | – | – |
| *Buccinum bayani* | 8 | B | – | – | + | + | + |
| *Buccinum cnismatum* | 8 | B | – | * | * | – | + |
| *Buccinum ectomycina* | 8 | B | – | – | + | + | – |
| *Buccinum fukureum* | 8 | B | – | – | + | – | – |
| *Buccinum glaciale* | 8 | B | * | + | + | – | + |
| *Buccinum kinukatsugai* | 8 | B | – | – | + | – | – |
| *Buccinum lischkeanum* | 8 | B | – | – | + | – | * |
| *Buccinum miyauchii* | 8 | B | – | – | + | – | – |
| *Buccinum nodocostum* | 8 | B | – | – | + | – | – |
| *Buccinum ochotense* | 8 | B | – | – | * | + | – |
| *Buccinum osagawai* | 8 | B | – | – | + | – | – |
| *Buccinum pemphigus* | 8 | B | – | * | + | – | + |
| *Buccinum plectrum* | 8 | B | * | + | * | – | * |
| *Buccinum polare* | 8 | B | + | + | + | – | * |
| *Buccinum polium* | 8 | B | – | – | + | – | – |
| *Buccinum rossicum* | 8 | B | – | + | + | + | + |
| *Buccinum scalariforme* | 8 | B | + | + | – | – | * |
| *Buccinum shiretokoensis* | 8 | B | – | – | + | – | – |
| *Buccinum striatellum* | 8 | B | – | – | * | + | – |
| *Buccinum striatissimum* | 8 | B | – | – | * | + | * |
| *Buccinum tenuisulcatum* | 8 | B | – | – | + | – | + |
| *Buccinum verkruzeni* | 8 | B | – | – | + | + | * |
| *Callanthias sp.* | 1 | B | – | – | – | – | + |
| Callionymidae gen. sp. | 1 | B | – | – | – | – | + |
| *Callista sp.* | 9 | B | – | * | * | + | * |
| *Callithaca adamsi* | 9 | B | – | * | * | + | * |
| *Calycopsis nematophora* | 14 | MB | – | + | + | – | + |
| Campanulinidae gen. sp. | 14 | B | – | * | – | – | + |
| *Cancer sp.* | 4 | B | – | * | – | * | + |
| *Caranx sexfasciatus* | 1 | M | – | – | – | – | + |
| Carapidae gen. sp. | 1 | M | – | – | – | – | + |
| *Careproctus acanthodes* | 1 | B | – | – | + | + | – |
| *Careproctus bathycoetus* | 1 | B | – | – | + | – | – |
| *Careproctus colletti* | 1 | MB | – | + | + | + | + |
| *Careproctus curilanus* | 1 | B | – | – | – | – | + |
| *Careproctus cyclocephalus* | 1 | MB | – | – | + | – | + |
| *Careproctus cypselurus* | 1 | MB | – | + | + | * | + |
| *Careproctus furcellus* | 1 | MB | – | + | + | – | + |
| *Careproctus gilberti* | 1 | B | – | + | – | – | * |
| *Careproctus homopterus* | 1 | M | – | – | + | – | – |
| *Careproctus macrodiscus* | 1 | MB | – | + | + | – | + |
| *Careproctus mederi* | 1 | MB | – | – | + | – | + |
| *Careproctus melanurus* | 1 | MB | – | + | + | – | + |
| *Careproctus mollis* | 1 | B | – | + | – | – | + |
| *Careproctus ostentum* | 1 | MB | – | * | + | – | * |
| *Careproctus phasma* | 1 | B | – | + | + | – | + |
| *Careproctus pycnosoma* | 1 | M | – | – | + | – | * |
| *Careproctus rastrinus* | 1 | MB | + | + | + | + | + |
| *Careproctus rhodomelas* | 1 | M | – | – | – | + | – |
| *Careproctus roseofuscus* | 1 | MB | – | + | + | + | + |
| *Careproctus scottae* | 1 | B | – | + | – | – | – |
| *Careproctus segaliensis* | 1 | B | – | – | + | – | – |
| *Careproctus simus* | 1 | B | – | * | * | – | + |
| *Careproctus sinensis* | 1 | MB | – | – | + | + | + |
| *Careproctus trachysoma* | 1 | MB | – | – | + | + | + |
| *Careproctus zachirus* | 1 | B | – | * | – | – | + |
| *Carinaria cristata* | 8 | M | – | – | – | – | + |
| *Carinaria japonica* | 8 | MB | – | – | – | – | + |
| *Caristius macropus* | 1 | MB | – | + | – | – | + |
| *Caryophyllia smithii* | 14 | B | – | – | + | – | * |
| *Caulophryne jordani* | 1 | B | – | – | – | – | + |
| *Caulophryne pelagica* | 1 | M | – | – | – | – | + |
| *Centrobranchus brevirostris* | 1 | M | – | – | – | – | + |
| *Centrophorus squamosus* | 1 | M | – | – | – | – | + |
| Centrophrynidae gen. sp. | 1 | B | – | – | – | – | + |
| *Centroscyllium ritteri* | 1 | MB | – | – | – | – | + |
| *Cephaloscyllium umbratile* | 1 | M | – | – | – | * | + |
| *Cepola schlegelii* | 1 | M | – | – | – | – | + |
| *Ceramaster japonicus* | 13 | B | – | + | + | – | * |
| *Ceramaster patagonicus productus* | 13 | B | – | * | + | – | * |
| *Ceratias holboelli* | 1 | MB | – | + | + | – | + |
| *Ceratoscopelus maderensis* | 1 | M | – | – | – | – | + |
| *Ceratoscopelus townsendi* | 1 | M | – | – | – | – | + |
| *Ceratoscopelus warmingii* | 1 | MB | – | – | + | – | + |
| *Cerebratulus sp.* | 20 | B | – | * | – | + | * |
| *Cetomimus sp.* | 1 | B | – | – | – | – | + |
| *Chaenophryne draco* | 1 | B | – | – | – | – | + |
| *Chaetopterus variopedatus* | 20 | B | – | * | + | * | * |
| *Charybdis japonica* | 4 | B | – | – | – | + | + |
| *Chascanopsetta prognatha* | 1 | B | – | – | – | – | + |
| *Chascanopsetta prorigera* | 1 | B | – | – | – | – | + |
| *Chauliodus macouni* | 1 | MB | – | + | + | – | + |
| *Chauliodus sloani* | 1 | MB | – | + | + | – | + |
| *Chaunax fimbriatus* | 1 | B | – | – | – | – | + |
| *Chaunax pictus* | 1 | B | – | – | – | – | + |
| *Cheilopogon pinnatibarbatus* | 1 | M | – | – | – | – | + |
| *Cheiraster dawsoni* | 13 | B | + | * | + | + | * |
| *Cheiraster tuberculatus* | 13 | B | – | – | – | + | – |
| *Chelonodon patoca* | 1 | M | – | – | – | – | + |
| *Chelyosoma inaequale* | 3 | B | + | – | * | + | * |
| *Chelyosoma orientale* | 3 | B | + | * | – | * | * |
| *Chesnonia verrucosa* | 1 | B | – | + | – | – | – |
| *Chiasmodon niger* | 1 | M | – | – | – | – | + |
| *Chilara taylori* | 1 | B | – | – | – | – | + |
| *Chilorhinus sp.* | 1 | M | – | – | – | – | + |
| *Chimaera owstoni* | 1 | B | – | – | – | – | + |
| *Chionoecetes angulatus* | 4 | B | – | + | + | * | + |
| *Chionoecetes bairdi* | 4 | B | – | + | + | * | + |
| *Chionoecetes japonicus* | 4 | B | – | – | – | + | * |
| *Chionoecetes opilio* | 4 | B | + | + | + | + | + |
| *Chionoecetes tanneri* | 4 | B | – | + | – | – | + |
| *Chiridota laevis* | 12 | B | + | + | + | + | + |
| *Chirolophis decoratus* | 1 | B | – | + | – | – | – |
| *Chirolophis japonicus* | 1 | B | – | – | + | + | – |
| *Chirolophis saitone* | 1 | B | – | – | – | + | – |
| *Chirolophis snyderi* | 1 | B | – | + | + | + | + |
| *Chirona evermanni* | 6 | B | – | + | + | + | + |
| *Chiroteuthis calyx* | 7 | MB | – | + | + | – | + |
| *Chlamys albida* | 9 | B | * | + | + | + | + |
| *Chlamys behringiana* | 9 | B | + | + | * | * | * |
| *Chlamys farreri nipponensis* | 9 | B | – | – | – | + | * |
| *Chlamys rosealbus* | 9 | B | – | – | + | + | + |
| *Chlamys rubida* | 9 | B | – | + | * | * | * |
| *Chlorophthalmus albatrossis* | 1 | B | – | – | – | – | + |
| *Chlorophthalmus proridens* | 1 | MB | – | – | – | – | + |
| *Chondrocladia gigantea* | 17 | B | – | * | + | – | + |
| *Chrysaora helvola* | 14 | M | + | – | * | – | – |
| *Chrysaora melanaster* | 14 | MB | + | + | + | + | + |
| *Chrysaora quinquecirrha* | 14 | MB | + | + | + | – | + |
| *Chtenopteryx sicula* | 7 | M | – | – | – | – | + |
| *Ciliatocardium ciliatum* | 9 | B | + | * | + | + | * |
| *Cistenides hyperborea* | 20 | B | * | + | – | – | * |
| *Citharichthys sordidus* | 1 | B | – | * | – | – | + |
| *Cladaster validus* | 13 | B | – | * | * | – | + |
| *Cladocarpus formosus* | 14 | B | – | – | – | – | + |
| *Cladorhiza bathycrinoides* | 17 | B | – | – | * | – | + |
| *Cleisthenes herzensteini* | 1 | MB | – | – | + | + | + |
| *Clidoderma asperrimum* | 1 | MB | – | + | + | + | + |
| *Clinocardium californiense* | 9 | B | * | + | + | + | * |
| *Clinocardium ciliatum* | 9 | B | + | + | + | + | * |
| *Clinocottus sp.* | 1 | B | – | – | – | – | + |
| *Clinopegma borealis* | 8 | B | – | – | + | – | * |
| *Clinopegma chikaoi* | 8 | B | – | – | + | – | – |
| *Clinopegma decora* | 8 | B | – | – | + | + | – |
| *Clinopegma magnum* | 8 | B | + | + | + | + | * |
| *Clinopegma ochotensis* | 8 | B | – | – | + | – | * |
| *Clio recurva* | 8 | M | – | – | – | – | + |
| *Clupea pallasii* | 1 | MB | + | + | + | + | + |
| *Coelorinchus gilberti* | 1 | MB | – | – | – | – | + |
| *Coelorinchus macrochir* | 1 | MB | – | – | * | – | + |
| *Coelorinchus matsubarai* | 1 | B | – | – | – | – | + |
| *Colobonema sp.* | 14 | M | – | – | – | – | + |
| *Cololabis saira* | 1 | MB | – | + | + | + | + |
| *Colus minor* | 8 | B | – | – | + | * | – |
| *Colus pubescens* | 8 | B | + | – | – | – | – |
| *Conger japonicus* | 1 | B | – | – | – | – | + |
| *Conger myriaster* | 1 | M | – | – | – | – | + |
| *Congriscus megastomus* | 1 | M | – | – | – | – | + |
| Convolutidae gen. sp. | 20 | B | – | – | + | – | * |
| *Cookeolus japonicus* | 1 | MB | – | – | – | – | + |
| *Corolla ovata* | 8 | M | – | – | – | – | + |
| *Coryphaena hippurus* | 1 | M | – | – | * | + | * |
| *Coryphaenoides acrolepis* | 1 | MB | – | + | + | – | + |
| *Coryphaenoides cinereus* | 1 | MB | – | + | + | – | + |
| *Coryphaenoides filifer* | 1 | B | – | – | – | – | + |
| *Coryphaenoides longifilis* | 1 | MB | – | + | + | – | + |
| *Coryphaenoides nasutus* | 1 | MB | – | – | – | – | + |
| *Cottiusculus gonez* | 1 | B | – | – | * | + | * |
| *Cottiusculus schmidti* | 1 | B | – | – | + | + | + |
| *Cottunculus microps* | 1 | B | + | – | – | – | – |
| *Cottus sp.* | 1 | B | – | – | – | + | – |
| *Cranchia scabra* | 7 | M | – | – | – | – | + |
| *Crangon alaskensis* | 5 | B | * | + | – | – | * |
| *Crangon dalli* | 5 | MB | + | + | + | + | + |
| *Crangon septemspinosa* | 5 | MB | * | + | + | + | + |
| *Crassostrea gigas* | 9 | B | – | – | – | + | * |
| *Crenomytilus grayanus* | 9 | B | – | – | + | + | + |
| *Crepidula derjugini* | 8 | B | – | – | – | + | – |
| *Crossaster borealis ochotensis* | 13 | B | – | + | + | * | * |
| *Crossaster papposus* | 13 | B | + | + | + | + | + |
| *Cryptacanthodes aleutensis* | 1 | B | – | + | – | – | + |
| *Cryptacanthodes bergi* | 1 | MB | – | – | + | + | – |
| *Cryptacanthodes giganteus* | 1 | B | – | * | – | – | + |
| *Cryptochiton stelleri* | 10 | B | – | – | + | + | + |
| *Cryptonatica affinis* | 8 | B | + | + | + | + | + |
| *Cryptonatica aleutica* | 8 | B | + | + | – | + | * |
| *Cryptonatica janthostoma* | 8 | B | – | * | + | + | * |
| *Cryptopsaras couesii* | 1 | M | – | – | – | – | + |
| *Crystallias matsushimae* | 1 | MB | – | + | + | + | + |
| *Crystallichthys cyclospilus* | 1 | MB | – | + | + | + | + |
| *Ctenodiscus crispatus* | 13 | B | + | + | + | + | + |
| *Cubiceps capensis* | 1 | B | – | – | – | – | + |
| *Cubiceps pauciradiatus* | 1 | M | – | – | – | – | + |
| *Cucumaria calcigera* | 12 | B | + | * | * | * | * |
| *Cucumaria fallax* | 12 | B | – | * | + | – | * |
| *Cucumaria frondosa japonica* | 12 | B | – | + | + | + | + |
| *Cucumaria glacialis* | 12 | B | + | * | * | * | – |
| *Cucumaria okhotensis* | 12 | B | – | – | + | – | – |
| *Cyanea capillata* | 14 | MB | + | + | + | + | + |
| *Cyclocardia crassidens* | 9 | B | * | + | + | * | * |
| *Cyclopsis tentacularis* | 1 | M | – | – | + | – | – |
| *Cyclopteropsis bergi* | 1 | MB | + | * | + | + | + |
| *Cyclopteropsis inarmatus* | 1 | B | – | * | + | – | – |
| *Cyclopteropsis lindbergi* | 1 | MB | – | + | + | + | – |
| *Cyclopteropsis phrynoides* | 1 | B | – | + | – | – | * |
| *Cyclopteropsis popovi* | 1 | MB | – | – | + | – | * |
| *Cyclosalpa sp.* | 3 | M | – | – | – | * | + |
| *Cycloteuthis akimushkini* | 7 | M | – | – | – | – | + |
| *Cyclothone alba* | 1 | M | – | – | – | – | + |
| *Cyclothone atraria* | 1 | MB | – | + | + | – | + |
| *Cyclothone pseudopallida* | 1 | M | – | + | – | – | * |
| *Cymatogaster aggregata* | 1 | B | – | – | – | – | + |
| *Dalatias licha* | 1 | MB | – | – | – | – | + |
| *Dasyatis akajei* | 1 | M | – | – | – | – | + |
| *Dasycottus setiger* | 1 | MB | – | + | + | + | + |
| *Davidijordania brachyrhyncha* | 1 | B | – | – | + | + | – |
| *Davidijordania lacertina* | 1 | B | – | – | – | + | – |
| *Decapterus kurroides* | 1 | M | – | – | – | – | + |
| *Decapterus muroadsi* | 1 | M | – | – | – | – | + |
| *Decapterus russelli* | 1 | M | – | – | – | – | + |
| *Delectopecten sp.* | 9 | B | – | * | * | + | * |
| *Dendrobeania flustroides* | 16 | B | + | * | * | * | + |
| *Dermaturus mandtii* | 4 | B | * | + | + | + | * |
| *Desmodema lorum* | 1 | M | – | – | – | – | + |
| *Desmodema polystictum* | 1 | M | – | – | – | – | + |
| *Diaphus adenomus* | 1 | M | – | – | – | – | + |
| *Diaphus anderseni* | 1 | M | – | – | – | – | + |
| *Diaphus brachycephalus* | 1 | M | – | – | – | – | + |
| *Diaphus chrysorhynchus* | 1 | M | – | – | – | – | + |
| *Diaphus effulgens* | 1 | M | – | – | – | – | + |
| *Diaphus fragilis* | 1 | M | – | – | – | – | + |
| *Diaphus gigas* | 1 | MB | – | – | – | – | + |
| *Diaphus kuroshio* | 1 | MB | – | – | – | – | + |
| *Diaphus metopoclampus* | 1 | M | – | – | – | – | + |
| *Diaphus mollis* | 1 | M | – | – | – | – | + |
| *Diaphus perspicillatus* | 1 | MB | – | – | – | – | + |
| *Diaphus regani* | 1 | M | – | – | – | – | + |
| *Diaphus richardsoni* | 1 | M | – | – | – | – | + |
| *Diaphus schmidti* | 1 | M | – | – | – | – | + |
| *Diaphus signatus* | 1 | M | – | – | – | – | + |
| *Diaphus theta* | 1 | MB | – | + | + | – | + |
| *Didemnum sp.* | 3 | B | * | * | – | + | * |
| Diodontidae gen. sp. | 1 | M | – | – | – | – | + |
| *Diogenichthys laternatus* | 1 | M | – | – | – | – | + |
| *Diplophos orientalis* | 1 | M | – | – | – | – | + |
| *Diplophos taenia* | 1 | M | – | – | – | – | + |
| *Diplopteraster multipes* | 13 | B | – | + | + | – | * |
| *Diplospinus multistriatus* | 1 | M | – | – | – | – | + |
| *Dipsacaster borealis* | 13 | B | – | + | + | – | * |
| *Diretmichthys parini* | 1 | M | – | – | – | – | + |
| *Diretmus sp.* | 1 | M | – | – | – | – | + |
| *Distolasterias elegans* | 13 | B | – | – | * | + | – |
| *Distolasterias nipon* | 13 | B | – | – | + | + | + |
| *Doederleinia berycoides* | 1 | M | – | – | – | – | + |
| *Dolicholagus longirostris* | 1 | M | – | – | – | – | + |
| *Dolichopteryx parini* | 1 | M | – | + | + | – | + |
| Doliolidae gen. sp. | 3 | M | – | * | – | * | + |
| *Doryteuthis opalescens* | 7 | B | – | – | – | – | + |
| *Echinarachnius parma* | 11 | B | + | + | + | + | + |
| *Echinocardium cordatum* | 11 | B | – | * | + | + | + |
| *Echiostoma barbatum* | 1 | M | – | – | – | – | + |
| *Echiurus echiurus* | 20 | B | + | * | * | + | + |
| *Elassochirus cavimanus* | 6 | B | – | + | + | * | * |
| *Elassochirus gilli* | 6 | B | – | + | + | – | + |
| *Elassodiscus obscurus* | 1 | MB | – | + | + | – | + |
| *Elassodiscus tremebundus* | 1 | MB | – | + | + | – | + |
| *Electrona risso* | 1 | MB | – | – | – | – | + |
| *Eleginus gracilis* | 1 | MB | + | + | + | + | + |
| *Embassichthys bathybius* | 1 | B | – | + | + | – | + |
| *Emmelichthys struhsakeri* | 1 | MB | – | – | – | – | + |
| *Engraulis japonicus* | 1 | MB | – | + | + | + | + |
| *Engraulis mordax* | 1 | B | – | – | – | – | + |
| *Ennucula tenuis* | 9 | B | * | + | – | * | * |
| *Enophrys diceraus* | 1 | MB | * | + | + | + | + |
| *Enophrys lucasi* | 1 | B | * | + | – | – | * |
| *Enoploteuthis chunii* | 7 | MB | – | – | – | + | + |
| *Enteroctopus dofleini* | 7 | MB | – | + | + | + | + |
| *Entosphenus tridentatus* | 2 | MB | – | + | + | – | + |
| *Eopsetta grigorjewi* | 1 | B | – | – | + | + | * |
| *Eopsetta jordani* | 1 | B | – | – | – | – | + |
| *Epigonus atherinoides* | 1 | B | – | – | – | – | + |
| *Epigonus denticulatus* | 1 | MB | – | – | – | – | + |
| *Eptatretus deani* | 2 | B | – | – | – | – | + |
| *Eptatretus stouti* | 2 | B | – | – | – | – | + |
| *Erimacrus isenbeckii* | 4 | B | * | + | + | + | + |
| *Eriocheir japonica* | 4 | B | – | – | – | + | * |
| *Errinopora stylifera* | 14 | B | – | – | + | * | * |
| *Erythrocles schlegelii* | 1 | B | – | – | – | – | + |
| *Etmopterus lucifer* | 1 | MB | – | – | – | – | + |
| *Etmopterus pusillus* | 1 | MB | – | – | – | – | + |
| *Etmopterus unicolor* | 1 | M | – | – | – | – | + |
| *Etrumeus micropus* | 1 | M | – | – | – | + | + |
| *Etrumeus teres* | 1 | M | – | – | – | * | + |
| *Eualus biunguis* | 5 | MB | – | + | + | + | + |
| *Eualus fabricii* | 5 | B | * | + | + | + | * |
| *Eualus gaimardii belcheri* | 5 | MB | + | + | + | + | * |
| *Eualus leptognathus* | 5 | B | – | – | + | * | * |
| *Eualus macilentus* | 5 | MB | + | + | + | + | * |
| *Eualus middendorffi* | 5 | B | – | – | + | + | * |
| *Eualus suckleyi* | 5 | MB | * | + | + | – | * |
| *Eualus townsendi* | 5 | MB | – | + | + | + | * |
| *Eucleoteuthis luminosa* | 7 | M | – | – | – | – | + |
| *Eumesogrammus praecisus* | 1 | MB | * | + | + | – | + |
| *Eumicrotremus andriashevi* | 1 | MB | * | + | + | – | – |
| *Eumicrotremus asperrimus* | 1 | MB | – | + | + | + | + |
| *Eumicrotremus derjugini* | 1 | MB | + | + | + | – | + |
| *Eumicrotremus orbis* | 1 | MB | + | + | + | + | + |
| *Eumicrotremus pacificus* | 1 | MB | – | + | + | + | + |
| *Eumicrotremus schmidti* | 1 | MB | – | – | + | – | – |
| *Eumicrotremus soldatovi* | 1 | MB | – | + | + | – | * |
| *Eumicrotremus taranetzi* | 1 | MB | – | + | + | + | + |
| *Eumicrotremus tartaricus* | 1 | MB | – | – | + | + | + |
| *Eunephthya sp.* | 14 | B | + | + | + | * | * |
| *Eunoe nodosa* | 20 | B | * | * | + | + | + |
| *Eupentacta fraudatrix* | 12 | B | – | – | – | + | * |
| *Euphrosine hortensis* | 20 | B | – | + | – | – | + |
| *Euprotomicrus bispinatus* | 1 | M | – | – | – | – | + |
| *Eurymen bassargini* | 1 | B | – | – | – | + | + |
| *Eurymen gyrinus* | 1 | MB | – | + | + | + | + |
| *Eurypharynx pelecanoides* | 1 | M | – | – | – | – | + |
| *Eusergestes similis* | 5 | MB | – | – | + | – | + |
| *Euspira pallida* | 8 | B | * | + | + | + | * |
| *Eustomias gibbsi* | 1 | M | – | – | – | – | + |
| *Evasterias echinosoma* | 13 | B | * | + | + | + | + |
| *Evasterias retifera* | 13 | B | – | * | + | + | * |
| *Evermannella sp.* | 1 | M | – | – | – | – | + |
| *Ezocallista brevisiphonata* | 9 | B | – | – | * | + | + |
| *Fistularia petimba* | 1 | M | – | – | – | – | + |
| *Flagellostomias boureei* | 1 | M | – | – | – | – | + |
| *Flustra foliacea* | 16 | B | + | * | * | – | – |
| *Flustra kurilensis* | 16 | B | – | – | – | – | + |
| *Forcepia uschakowi* | 17 | B | – | – | + | – | – |
| *Freemanichthys thompsoni* | 1 | MB | – | + | + | + | + |
| *Fusitriton oregonensis* | 8 | B | – | + | + | + | + |
| *Gadus macrocephalus* | 1 | MB | + | + | + | + | + |
| Galatheidae gen. sp. | 6 | B | – | * | * | * | + |
| *Galeorhinus galeus* | 1 | B | – | – | – | – | + |
| *Galiteuthis phyllura* | 7 | MB | – | + | + | – | + |
| *Gasterosteus aculeatus* | 1 | MB | – | + | + | + | + |
| *Gempylus serpens* | 1 | MB | – | – | – | – | + |
| *Geodia cydonium* | 17 | B | – | – | – | + | – |
| *Gersemia fruticosa* | 14 | B | + | – | – | – | * |
| *Gersemia rubiformis* | 14 | B | + | + | + | + | * |
| *Gigantactis elsmani* | 1 | M | – | – | + | – | – |
| *Giganthias immaculatus* | 1 | M | – | – | – | – | + |
| Giganturidae gen. sp. | 1 | B | – | – | – | – | + |
| *Gilbertidia pustulosa* | 1 | B | – | + | + | – | + |
| *Glebocarcinus amphioetus* | 4 | B | – | – | – | + | * |
| *Glossanodon semifasciatus* | 1 | M | – | – | – | – | + |
| *Glyptocephalus stelleri* | 1 | MB | – | + | + | + | + |
| *Glyptocephalus zachirus* | 1 | MB | – | + | – | – | + |
| *Gnathophis nystromi* | 1 | MB | – | – | – | – | + |
| *Golfingia margaritacea* | 20 | B | + | * | + | + | * |
| *Gonatopsis japonicus* | 7 | MB | – | + | + | + | + |
| *Gonatopsis makko* | 7 | MB | – | + | + | * | + |
| *Gonatopsis octopedatus* | 7 | MB | – | + | + | + | + |
| *Gonatus berryi* | 7 | MB | – | + | + | – | + |
| *Gonatus californiensis* | 7 | M | – | + | – | – | * |
| *Gonatus kamtschaticus* | 7 | MB | + | + | + | – | + |
| *Gonatus madokai* | 7 | MB | – | + | + | + | + |
| *Gonatus onyx* | 7 | MB | + | + | + | – | + |
| *Gonatus pyros* | 7 | MB | – | + | + | – | + |
| *Gonatus tinro* | 7 | MB | – | + | + | – | + |
| *Gonorhynchus gonorhynchus* | 1 | B | – | – | – | – | + |
| *Gonostoma sp.* | 1 | M | – | – | – | – | + |
| *Gorgonocephalus eucnemis* | 13 | B | + | + | + | + | + |
| Grammatidae gen. sp. | 1 | B | – | – | – | – | + |
| *Grammatonotus laysanus* | 1 | B | – | – | – | – | + |
| *Grimalditeuthis bonplandii* | 7 | M | – | – | – | – | + |
| *Gymnelopsis brashnikovi* | 1 | B | – | – | + | – | – |
| *Gymnelopsis brevifenestrata* | 1 | B | – | – | + | – | – |
| *Gymnelopsis ocellatus* | 1 | B | – | – | + | – | + |
| *Gymnelopsis ochotensis* | 1 | B | – | – | + | – | – |
| *Gymnelus pauciporus* | 1 | B | – | + | * | – | * |
| *Gymnelus viridis* | 1 | B | * | + | – | – | – |
| *Gymnocanthus detrisus* | 1 | MB | – | + | + | + | + |
| *Gymnocanthus galeatus* | 1 | MB | + | + | + | + | + |
| *Gymnocanthus herzensteini* | 1 | MB | – | + | + | + | + |
| *Gymnocanthus intermedius* | 1 | B | – | – | + | + | + |
| *Gymnocanthus pistilliger* | 1 | MB | + | + | + | + | + |
| *Gymnocanthus tricuspis* | 1 | MB | + | + | – | – | – |
| *Gymnura japonica* | 1 | M | – | – | – | – | + |
| *Hadropareia middendorffii* | 1 | B | – | – | + | – | – |
| *Halaelurus buergeri* | 1 | M | – | – | – | – | + |
| *Halargyreus johnsonii* | 1 | MB | – | + | + | – | + |
| *Halecium muricatum* | 14 | B | – | * | – | * | + |
| *Halieutaea stellata* | 1 | B | – | – | – | – | + |
| *Haliphron atlanticus* | 7 | MB | – | + | + | – | + |
| *Halocynthia aurantium* | 3 | B | + | + | + | + | + |
| *Halocynthia roretzi* | 3 | B | – | – | – | + | * |
| *Halosaurus sp.* | 1 | B | – | – | – | – | + |
| *Hapalogaster dentata* | 4 | B | – | + | + | + | * |
| *Hapalogaster grebnitzkii* | 4 | B | * | + | + | + | + |
| *Harmothoe imbricata* | 20 | B | * | * | * | * | + |
| *Harriotta raleighana* | 1 | M | – | – | – | – | + |
| *Helicocranchia pfefferi* | 7 | MB | – | – | – | – | + |
| *Helicolenus avius* | 1 | B | – | – | – | – | + |
| *Helicolenus fedorovi* | 1 | B | – | – | – | – | + |
| *Helicolenus hilgendorfii* | 1 | B | – | – | – | * | + |
| *Helicosalpa sp.* | 3 | M | – | – | – | – | + |
| *Heliometra glacialis* | 13 | B | * | + | + | + | + |
| *Hemigrapsus penicillatus* | 4 | B | – | – | * | + | * |
| *Hemigrapsus sanguineus* | 4 | B | – | – | * | + | * |
| *Hemilepidotus gilberti* | 1 | MB | + | + | + | + | + |
| *Hemilepidotus hemilepidotus* | 1 | MB | – | + | + | – | + |
| *Hemilepidotus jordani* | 1 | MB | + | + | + | + | + |
| *Hemilepidotus papilio* | 1 | MB | + | + | + | – | + |
| *Hemilepidotus spinosus* | 1 | B | – | + | – | – | + |
| *Hemilepidotus zapus* | 1 | B | – | * | – | – | + |
| *Hemitripterus bolini* | 1 | MB | – | + | * | – | + |
| *Hemitripterus villosus* | 1 | MB | – | + | + | + | + |
| *Henricia aspera* | 13 | B | – | * | + | + | * |
| *Henricia leviuscula* | 13 | B | – | * | * | + | * |
| *Henricia leviuscula spiculifera* | 13 | B | – | * | – | – | + |
| *Henricia reticulata* | 13 | B | – | – | – | + | – |
| *Heptacarpus flexus* | 5 | B | – | + | * | + | * |
| Heteroporidae gen. sp. | 16 | B | * | * | + | * | * |
| *Hexagrammos decagrammus* | 1 | B | – | + | – | – | + |
| *Hexagrammos lagocephalus* | 1 | MB | – | + | + | + | + |
| *Hexagrammos octogrammus* | 1 | MB | – | + | + | + | + |
| *Hexagrammos otakii* | 1 | B | – | – | + | + | + |
| *Hexagrammos stelleri* | 1 | MB | + | + | + | + | + |
| *Hexanchus griseus* | 1 | B | – | – | – | – | + |
| *Hiatella arctica* | 9 | B | * | * | * | + | * |
| *Himantolophus groenlandicus* | 1 | B | – | – | – | – | + |
| *Hippasteria phrygiana* | 13 | B | – | + | + | * | + |
| *Hippocampus mohnikei* | 1 | MB | – | – | – | + | – |
| *Hippoglossina stomata* | 1 | B | – | + | – | – | + |
| *Hippoglossoides dubius* | 1 | MB | – | – | + | + | + |
| *Hippoglossoides elassodon* | 1 | MB | – | + | + | + | + |
| *Hippoglossoides robustus* | 1 | MB | + | + | + | + | + |
| *Hippoglossus stenolepis* | 1 | MB | + | + | + | + | + |
| *Histiobranchus bathybius* | 1 | M | – | * | – | – | + |
| *Histioteuthis hoylei* | 7 | MB | – | + | * | – | + |
| *Histioteuthis meleagroteuthis* | 7 | M | – | – | – | – | + |
| *Holtbyrnia innesi* | 1 | MB | – | + | + | – | + |
| *Homaxinella subdola* | 17 | B | – | * | * | + | * |
| *Hoplostethus crassispinus* | 1 | B | – | – | – | – | + |
| *Hormiphora cucumis* | 15 | M | * | * | – | + | + |
| *Hormiphora palmata* | 15 | M | – | – | * | * | + |
| *Howella parini* | 1 | MB | – | – | – | – | + |
| *Howella sherborni* | 1 | M | – | – | – | – | + |
| *Howella zina* | 1 | B | – | – | – | – | + |
| *Hozukius guyotensis* | 1 | B | – | – | – | – | + |
| *Hyaloteuthis pelagica* | 7 | M | – | – | – | – | + |
| *Hyas alutaceus* | 4 | B | * | * | + | * | * |
| *Hyas coarctatus* | 4 | B | + | + | + | + | + |
| *Hyas lyratus* | 4 | B | * | + | * | – | * |
| *Hydrolagus barbouri* | 1 | MB | – | – | – | * | + |
| *Hydrolagus colliei* | 1 | B | – | – | – | – | + |
| *Hydrolagus purpurescens* | 1 | M | – | – | * | – | + |
| *Hygophum proximum* | 1 | M | – | – | – | – | + |
| *Hygophum reinhardtii* | 1 | M | – | – | – | – | + |
| *Hymenodora frontalis* | 5 | MB | – | * | + | – | + |
| *Hyperoglyphe japonica* | 1 | MB | – | – | + | + | + |
| *Hypomesus japonicus* | 1 | MB | – | + | + | + | + |
| *Hypomesus nipponensis* | 1 | B | – | – | * | + | – |
| *Hypomesus olidus* | 1 | MB | * | + | + | * | + |
| *Hypoptychus dybowskii* | 1 | B | – | – | + | + | * |
| *Hypsagonus corniger* | 1 | B | – | – | * | + | – |
| *Hypsagonus quadricornis* | 1 | B | * | + | + | + | + |
| *Icelinus filamentosus* | 1 | B | – | – | – | – | + |
| *Icelinus tenuis* | 1 | B | – | – | – | – | + |
| *Icelus armatus* | 1 | B | – | – | + | – | – |
| *Icelus canaliculatus* | 1 | B | – | + | + | + | + |
| *Icelus cataphractus* | 1 | MB | – | – | + | + | + |
| *Icelus gilberti* | 1 | B | – | – | + | + | – |
| *Icelus perminovi* | 1 | B | – | – | + | – | + |
| *Icelus rastrinoides* | 1 | B | – | – | – | + | – |
| *Icelus spatula* | 1 | MB | + | + | + | – | + |
| *Icelus spiniger* | 1 | MB | – | + | + | + | + |
| *Icelus stenosomus* | 1 | MB | – | – | – | + | + |
| *Icelus uncinalis* | 1 | B | – | + | + | – | – |
| *Ichthyococcus elongatus* | 1 | M | – | – | – | – | + |
| *Icosteus aenigmaticus* | 1 | MB | – | + | + | – | + |
| *Idiacanthus antrostomus* | 1 | MB | – | – | – | – | + |
| *Idiacanthus fasciola* | 1 | M | – | – | – | – | + |
| *Idothea sp.* | 6 | B | – | – | – | * | + |
| *Iridoteuthis iris* | 7 | MB | – | – | – | – | + |
| *Isistius brasiliensis* | 1 | M | – | – | – | – | + |
| *Isodictya rigida* | 17 | B | – | * | + | – | + |
| *Isopsetta isolepis* | 1 | B | – | + | – | – | + |
| *Isurus oxyrinchus* | 1 | M | – | – | – | + | + |
| *Japetella diaphana* | 7 | MB | – | + | + | – | + |
| *Kajikia audax* | 1 | M | – | – | – | – | + |
| *Kali indica* | 1 | M | – | – | – | – | + |
| *Kareius bicoloratus* | 1 | B | – | – | * | + | * |
| *Kasatkia memorabilis* | 1 | B | – | – | + | + | – |
| *Katsuwonus pelamis* | 1 | M | – | – | – | * | + |
| *Konosirus punctatus* | 1 | MB | – | – | – | + | – |
| *Krusensterniella maculata* | 1 | B | – | – | – | + | – |
| *Labidochirus splendescens* | 6 | B | * | * | + | – | * |
| *Labracoglossa argenteiventris* | 1 | M | – | – | – | – | + |
| *Lactoria diaphana* | 1 | M | – | – | – | – | + |
| *Laemonema longipes* | 1 | MB | – | + | + | + | + |
| *Lagocephalus lagocephalus* | 1 | M | – | – | – | – | + |
| *Lagocephalus wheeleri* | 1 | M | – | – | – | – | + |
| *Lamna ditropis* | 1 | MB | – | + | + | + | + |
| *Lampadena luminosa* | 1 | MB | – | – | – | – | + |
| *Lampadena urophaos* | 1 | MB | – | – | – | – | + |
| *Lampadena yaquinae* | 1 | MB | – | – | – | – | + |
| *Lampadioteuthis megaleia* | 7 | M | – | – | – | – | + |
| *Lampanyctus acanthurus* | 1 | M | – | – | – | – | + |
| *Lampanyctus alatus* | 1 | M | – | – | – | – | + |
| *Lampanyctus festivus* | 1 | M | – | – | – | – | + |
| *Lampanyctus jordani* | 1 | MB | – | + | + | – | + |
| *Lampanyctus nobilis* | 1 | M | – | – | – | – | + |
| *Lampanyctus simulator* | 1 | M | – | – | – | – | + |
| *Lampanyctus steinbecki* | 1 | M | – | – | – | – | + |
| *Lampanyctus tenuiformis* | 1 | M | – | – | – | – | + |
| *Lampanyctus turneri* | 1 | M | – | – | – | – | + |
| *Lampris guttatus* | 1 | M | – | – | + | – | + |
| *Laqueus californicus* | 19 | B | – | * | + | * | * |
| *Lateolabrax japonicus* | 1 | M | – | – | – | * | + |
| *Leachia pacifica* | 7 | M | – | – | – | – | + |
| *Lebbeus brandti* | 5 | MB | – | – | + | + | * |
| *Lebbeus grandimanus* | 5 | B | – | * | – | + | * |
| *Lebbeus groenlandicus* | 5 | MB | * | + | + | + | + |
| *Lebbeus heterochaelus* | 5 | B | – | – | + | – | – |
| *Lebbeus longidactylus* | 5 | B | – | + | + | – | – |
| *Lebbeus polaris* | 5 | B | + | * | + | – | * |
| *Lebbeus schrencki* | 5 | B | – | + | + | + | * |
| *Lebbeus unalaskensis* | 5 | B | – | * | + | * | – |
| *Lebbeus uschakovi* | 5 | B | – | – | + | – | – |
| *Leieschara orientalis* | 16 | B | – | * | * | * | + |
| *Lepas anatifera* | 6 | B | – | * | – | + | * |
| *Lepidion inosimae* | 1 | M | – | – | – | – | + |
| *Lepidion schmidti* | 1 | M | – | * | * | – | + |
| *Lepidocybium flavobrunneum* | 1 | M | – | – | – | – | + |
| *Lepidopsetta mochigarei* | 1 | MB | – | – | + | + | + |
| *Lepidopsetta polyxystra* | 1 | MB | – | + | + | – | + |
| *Lepidopus calcar* | 1 | MB | – | – | – | – | + |
| *Lepidotrigla japonica* | 1 | M | – | – | – | – | + |
| *Lepidotrigla microptera* | 1 | B | – | – | * | + | – |
| *Leptagonus decagonus* | 1 | MB | * | + | + | * | + |
| *Leptasterias arctica arctica* | 13 | B | + | + | – | – | + |
| *Leptasterias fisheri fisheri* | 13 | B | – | * | – | + | + |
| *Leptasterias orientalis japonica* | 13 | B | – | – | * | + | * |
| *Leptasterias polaris ushakovi* | 13 | B | + | + | – | – | * |
| *Leptochiton sp.* | 10 | B | – | + | * | * | * |
| *Leptoclinus maculatus* | 1 | MB | + | + | + | + | + |
| *Leptocottus armatus* | 1 | B | – | + | – | – | + |
| *Leptostomias gladiator* | 1 | M | – | – | – | – | + |
| *Leptostomias multifilis* | 1 | M | – | – | – | – | + |
| *Leptostomias robustus* | 1 | M | – | – | – | – | + |
| *Leptychaster propinquus* | 13 | B | – | * | + | + | * |
| *Lestidiops jayakari* | 1 | M | – | – | – | – | + |
| *Lestidiops ringens* | 1 | MB | – | + | + | – | + |
| *Lestidiops sphyraenopsis* | 1 | M | – | – | + | – | + |
| *Lestidium prolixum* | 1 | M | – | – | – | – | + |
| *Lestrolepis intermedia* | 1 | M | – | – | – | – | + |
| *Lestrolepis japonica* | 1 | M | – | – | – | – | + |
| *Lethasterias fusca* | 13 | B | – | – | * | + | * |
| *Lethasterias nanimensis* | 13 | B | * | * | + | + | * |
| *Lethenteron camtschaticum* | 2 | MB | + | + | + | + | + |
| *Lethotremus awae* | 1 | M | – | – | – | * | + |
| *Leucosyrinx circinata* | 8 | B | – | * | – | – | + |
| *Leuroglossus schmidti* | 1 | MB | – | + | + | – | + |
| *Limanda aspera* | 1 | MB | * | + | + | + | + |
| *Limanda proboscidea* | 1 | MB | * | + | + | – | + |
| *Limanda punctatissima* | 1 | MB | – | – | + | + | + |
| *Limanda sakhalinensis* | 1 | MB | + | + | + | + | + |
| *Limopsis uwadokoi* | 9 | B | – | – | – | – | + |
| Linophrynidae gen. sp. | 1 | M | – | – | – | – | + |
| *Liocranchia reinhardti* | 7 | MB | – | – | – | – | + |
| *Liopsetta glacialis* | 1 | MB | * | + | + | – | + |
| *Liopsetta pinnifasciata* | 1 | B | – | – | * | + | – |
| *Liparis agassizii* | 1 | MB | – | – | + | + | + |
| *Liparis brashnikovi* | 1 | B | – | – | – | + | * |
| *Liparis bristolensis* | 1 | B | * | + | – | – | – |
| *Liparis callyodon* | 1 | MB | – | + | – | – | * |
| *Liparis curilensis* | 1 | MB | – | – | + | – | – |
| *Liparis cyclopus* | 1 | MB | – | + | – | – | + |
| *Liparis dennyi* | 1 | B | – | + | – | – | * |
| *Liparis eos* | 1 | MB | – | + | + | * | – |
| *Liparis fabricii* | 1 | MB | + | – | – | – | – |
| *Liparis frenatus* | 1 | B | – | – | + | + | – |
| *Liparis gibbus* | 1 | MB | + | + | + | – | + |
| *Liparis latifrons* | 1 | MB | – | * | + | + | – |
| *Liparis megacephalus* | 1 | B | – | + | – | – | * |
| *Liparis mucosus* | 1 | B | – | + | – | – | – |
| *Liparis ochotensis* | 1 | MB | – | – | + | + | + |
| *Liparis owstoni* | 1 | B | – | – | – | + | * |
| *Liparis punctatus* | 1 | MB | – | – | + | + | + |
| *Liparis punctulatus* | 1 | B | – | – | – | + | – |
| *Liparis rhodosoma* | 1 | B | – | – | + | – | – |
| *Liparis rutteri* | 1 | B | – | + | – | – | – |
| *Liparis schantarensis* | 1 | B | – | – | + | – | * |
| *Liparis tanakae* | 1 | MB | – | – | + | + | + |
| *Liparis tessellatus* | 1 | MB | – | – | + | + | + |
| *Liparis tunicatus* | 1 | B | – | + | – | – | – |
| *Lipolagus ochotensis* | 1 | MB | – | + | + | + | + |
| *Liponema brevicorne* | 14 | B | – | * | + | – | * |
| *Lithodes aequispinus* | 4 | B | – | + | + | – | + |
| *Lithodes couesi* | 4 | B | – | + | + | – | + |
| *Liza haematocheila* | 1 | MB | – | – | * | + | * |
| *Lobianchia gemellarii* | 1 | M | – | – | – | – | + |
| *Loligo sp.* | 7 | M | – | – | – | – | + |
| *Lophaster furcilliger* | 13 | B | – | * | + | * | * |
| *Lophiodes miacanthus* | 1 | B | – | – | – | – | + |
| *Lophiomus sp.* | 1 | M | – | – | + | – | – |
| *Lophius litulon* | 1 | B | – | – | – | + | * |
| *Lophotus capellei* | 1 | M | – | – | – | – | + |
| *Lophotus lacepede* | 1 | M | – | – | – | – | + |
| *Lottia kogamogai* | 8 | B | – | – | – | + | * |
| *Luidia quinaria* | 13 | B | – | – | – | + | * |
| *Luidia quinaria bispinosa* | 13 | B | – | – | – | + | – |
| *Lumpenella longirostris* | 1 | MB | – | + | + | + | + |
| *Lumpenopsis pavlenkoi* | 1 | B | – | – | + | + | * |
| *Lumpenus fabricii* | 1 | MB | + | + | + | * | + |
| *Lumpenus sagitta* | 1 | MB | + | + | + | + | + |
| *Lussivolutopsius emphaticus* | 8 | B | – | – | + | + | * |
| *Lussivolutopsius furukawai* | 8 | B | – | – | + | * | – |
| *Lussivolutopsius hydractiniferus* | 8 | B | – | – | + | – | – |
| *Lussivolutopsius marinae* | 8 | B | – | – | + | + | * |
| *Lussivolutopsius ochotensis* | 8 | B | – | – | + | – | – |
| *Luvarus imperialis* | 1 | M | – | – | – | – | + |
| *Lycenchelys albomaculata* | 1 | B | – | – | + | – | * |
| *Lycenchelys camchatica* | 1 | B | – | + | + | – | * |
| *Lycenchelys crotalinus* | 1 | B | – | + | – | – | – |
| *Lycenchelys hippopotamus* | 1 | B | – | * | + | – | * |
| *Lycenchelys melanostomias* | 1 | B | – | – | + | – | – |
| *Lycenchelys ratmanovi* | 1 | B | – | + | – | – | * |
| *Lycodapus fierasfer* | 1 | MB | – | + | – | – | * |
| *Lycodapus microchir* | 1 | B | – | * | + | – | * |
| *Lycodapus poecilus* | 1 | B | – | * | + | – | – |
| *Lycodes albolineatus* | 1 | MB | – | – | + | – | + |
| *Lycodes albonotatus* | 1 | B | – | – | + | + | + |
| *Lycodes beringi* | 1 | B | + | + | * | – | + |
| *Lycodes brashnikovi* | 1 | B | – | – | + | – | – |
| *Lycodes brevipes* | 1 | MB | * | + | – | + | + |
| *Lycodes brunneofasciatus* | 1 | MB | – | + | + | + | + |
| *Lycodes caudimaculatus* | 1 | B | – | – | – | – | + |
| *Lycodes concolor* | 1 | MB | – | + | + | + | + |
| *Lycodes cortezianus* | 1 | B | – | – | – | – | + |
| *Lycodes diapterus* | 1 | MB | – | + | + | + | + |
| *Lycodes fasciatus* | 1 | MB | – | + | + | + | + |
| *Lycodes heinemanni* | 1 | B | – | – | + | + | – |
| *Lycodes hubbsi* | 1 | B | – | – | + | – | + |
| *Lycodes japonicus* | 1 | B | – | – | – | + | + |
| *Lycodes knipowitschi* | 1 | B | – | – | + | – | + |
| *Lycodes macrochir* | 1 | MB | – | – | + | – | – |
| *Lycodes macrolepis* | 1 | MB | – | – | + | + | – |
| *Lycodes microlepidotus* | 1 | B | – | – | + | – | – |
| *Lycodes microporus* | 1 | B | – | – | + | – | – |
| *Lycodes mucosus* | 1 | MB | + | + | + | – | – |
| *Lycodes nakamurae* | 1 | B | – | – | + | + | + |
| *Lycodes pacificus* | 1 | B | – | – | + | – | * |
| *Lycodes palearis* | 1 | MB | + | + | + | – | + |
| *Lycodes paucilepidotus* | 1 | B | – | – | + | – | – |
| *Lycodes pectoralis* | 1 | MB | – | – | + | – | + |
| *Lycodes polaris* | 1 | MB | + | + | – | – | – |
| *Lycodes raridens* | 1 | MB | + | + | + | + | + |
| *Lycodes rossi* | 1 | B | + | – | – | – | – |
| *Lycodes schmidti* | 1 | MB | – | – | + | + | – |
| *Lycodes semenovi* | 1 | MB | – | – | + | – | – |
| *Lycodes sigmatoides* | 1 | MB | – | + | + | + | * |
| *Lycodes soldatovi* | 1 | MB | – | + | + | – | + |
| *Lycodes tanakae* | 1 | MB | – | – | + | + | + |
| *Lycodes toyamensis* | 1 | B | + | – | + | + | – |
| *Lycodes turneri* | 1 | MB | + | + | – | – | – |
| *Lycodes uschakovi* | 1 | MB | – | – | + | + | + |
| *Lycodes yamatoi* | 1 | B | – | – | + | + | – |
| *Lycodes ygreknotatus* | 1 | MB | – | – | + | – | – |
| *Lycogrammoides schmidti* | 1 | MB | – | – | + | – | + |
| *Lycoteuthis sp.* | 7 | M | – | – | – | – | + |
| *Lycozoarces regani* | 1 | MB | – | – | + | + | + |
| *Lyopsetta exilis* | 1 | B | – | * | – | – | + |
| *Lysastrosoma anthosticta* | 13 | B | – | – | * | + | * |
| *Macoma calcarea* | 9 | B | * | + | * | + | * |
| *Macoma scarlatoi* | 9 | B | – | – | – | + | – |
| *Macropinna microstoma* | 1 | MB | – | + | + | – | + |
| *Macrorhamphosus scolopax* | 1 | MB | – | – | – | – | + |
| *Macrostomias sp.* | 1 | M | – | – | – | – | + |
| *Macrouroides inflaticeps* | 1 | B | – | – | – | – | + |
| *Mactra chinensis* | 9 | B | – | – | + | + | * |
| *Mactromeris polynyma* | 9 | B | * | * | + | * | * |
| *Magnisudis atlantica* | 1 | MB | – | + | + | – | + |
| *Makaira nigricans* | 1 | M | – | – | – | – | + |
| *Malacocephalus sp.* | 1 | B | – | – | – | – | + |
| *Malacocottus gibber* | 1 | M | – | – | + | * | + |
| *Malacocottus kincaidi* | 1 | B | – | + | + | – | + |
| *Malacocottus zonurus* | 1 | MB | – | + | + | + | + |
| *Malacosteus niger* | 1 | MB | – | * | – | – | + |
| Maldanidae gen. sp. | 20 | B | * | * | * | * | + |
| *Mallotus villosus catervarius* | 1 | MB | + | + | + | + | + |
| *Margarites costalis* | 8 | B | + | * | * | * | * |
| *Margrethia obtusirostra* | 1 | M | – | – | – | – | + |
| *Marukawichthys ambulator* | 1 | B | – | – | – | – | + |
| *Mastigoteuthis glaukopis* | 7 | M | – | – | – | – | + |
| *Maulisia argipalla* | 1 | M | – | – | – | – | + |
| *Maurolicus japonicus* | 1 | MB | – | – | – | + | + |
| *Meadia abyssalis* | 1 | B | – | – | – | – | + |
| *Meator rubatra* | 14 | M | – | + | + | – | + |
| *Megalocottus platycephalus* | 1 | MB | * | + | + | + | + |
| *Megalocranchia sp.* | 7 | MB | – | – | – | – | + |
| *Megayoldia thraciaeformis* | 9 | B | – | * | + | + | * |
| *Melamphaes lugubris* | 1 | MB | – | + | + | – | + |
| *Melanocetus johnsonii* | 1 | B | – | – | – | – | + |
| *Melanocetus murrayi* | 1 | MB | – | – | + | – | + |
| *Melanolagus berycoides* | 1 | MB | – | – | – | – | + |
| *Melanonus zugmayeri* | 1 | B | – | – | – | – | + |
| *Melanostomias pauciradius* | 1 | M | – | – | – | – | + |
| *Mercenaria stimpsoni* | 9 | B | – | – | * | + | * |
| *Merluccius productus* | 1 | B | – | – | – | – | + |
| *Mesenteripora meandrina* | 16 | B | – | – | – | – | + |
| *Mesocentrotus nudus* | 11 | B | – | – | + | + | * |
| *Mesocrangon intermedia* | 5 | MB | * | + | + | + | * |
| *Metacarcinus magister* | 4 | B | – | * | – | – | + |
| *Metacrangon robusta* | 5 | MB | – | – | + | + | – |
| *Metridium senile* | 14 | B | – | * | + | + | * |
| *Microciona lambei* | 17 | B | – | – | * | * | + |
| *Microcottus sellaris* | 1 | B | * | + | + | + | + |
| *Microgadus proximus* | 1 | B | – | + | – | – | + |
| *Microstomus achne* | 1 | B | – | – | + | * | + |
| *Microstomus pacificus* | 1 | B | – | + | * | – | + |
| *Microstomus shuntovi* | 1 | B | – | – | – | – | + |
| *Mizuhopecten yessoensis* | 9 | B | – | – | + | + | + |
| *Modiolus modiolus* | 9 | B | * | * | * | + | + |
| *Mola mola* | 1 | M | – | – | + | + | + |
| *Molgula griffithsii* | 3 | B | * | * | + | * | * |
| *Molpadia roretzii* | 12 | B | – | * | + | + | * |
| *Moroteuthis loennbergii* | 7 | M | – | – | – | – | + |
| *Moroteuthis robusta* | 7 | MB | – | + | + | – | + |
| *Mugil cephalus* | 1 | MB | – | – | – | + | – |
| *Musculus discors* | 9 | B | * | + | + | + | + |
| *Musculus glacialis* | 9 | B | * | * | – | * | + |
| *Musculus niger* | 9 | B | + | + | + | + | * |
| *Mya arenaria* | 9 | B | * | + | + | + | * |
| *Mya truncata* | 9 | B | * | * | + | + | * |
| *Mycale loveni* | 17 | B | * | * | + | – | + |
| *Myctophum asperum* | 1 | MB | – | – | – | – | + |
| *Myctophum lychnobium* | 1 | M | – | – | – | – | + |
| *Myctophum selenops* | 1 | M | – | – | – | – | + |
| *Myctophum spinosum* | 1 | M | – | – | – | – | + |
| *Myctophum nitidulum* | 1 | M | – | – | – | – | + |
| *Myoxocephalus brandtii* | 1 | B | – | * | + | + | + |
| *Myoxocephalus jaok* | 1 | MB | + | + | + | + | + |
| *Myoxocephalus niger* | 1 | B | – | * | + | * | – |
| *Myoxocephalus ochotensis* | 1 | MB | – | – | + | – | – |
| *Myoxocephalus polyacanthocephalus* | 1 | MB | + | + | + | + | + |
| *Myoxocephalus quadricornis* | 1 | B | * | + | + | – | – |
| *Myoxocephalus scorpioides* | 1 | B | – | + | + | – | – |
| *Myoxocephalus scorpius* | 1 | MB | + | + | – | – | * |
| *Myoxocephalus stelleri* | 1 | MB | – | + | + | + | + |
| *Myoxocephalus tuberculatus* | 1 | MB | – | – | + | – | + |
| *Myoxocephalus verrucosus* | 1 | MB | + | + | + | + | + |
| *Mytilus edulis* | 9 | B | * | * | * | + | * |
| *Mytilus trossulus* | 9 | B | * | * | + | + | * |
| *Myxilla incrustans* | 17 | B | * | + | * | * | + |
| *Myxoderma sacculatum* | 13 | B | – | * | + | – | * |
| *Nannobrachium nigrum* | 1 | MB | – | – | – | – | + |
| *Nannobrachium regale* | 1 | MB | – | + | + | – | + |
| *Nannobrachium ritteri* | 1 | M | – | * | – | – | + |
| *Nansenia candida* | 1 | M | – | + | – | – | + |
| *Naso unicornis* | 1 | M | – | – | – | – | + |
| *Natica sp.* | 8 | B | * | * | – | + | * |
| *Naucrates ductor* | 1 | M | – | – | – | – | + |
| *Nautichthys pribilovius* | 1 | MB | * | + | + | + | + |
| *Nautichthys robustus* | 1 | B | – | + | – | – | – |
| *Nealotus tripes* | 1 | M | – | – | – | – | + |
| *Neancistrolepis beringianus* | 8 | B | – | * | + | – | * |
| *Neancistrolepis glabra* | 8 | B | – | – | + | – | – |
| *Nectoliparis pelagicus* | 1 | MB | * | * | + | – | + |
| *Nematolampas regalis* | 7 | M | – | – | – | – | + |
| *Nemichthys scolopaceus* | 1 | MB | – | – | + | – | + |
| *Neocrangon communis* | 5 | MB | + | + | + | + | * |
| *Neognathophausia gigas* | 10 | B | – | * | – | – | + |
| *Neomenia yamamatoi* | 10 | B | – | + | * | – | * |
| *Neonesthes capensis* | 1 | M | – | – | – | – | + |
| *Neotrygon kuhlii* | 1 | M | – | – | – | – | + |
| *Nephtys sp.* | 20 | B | * | * | + | * | * |
| *Neptunea amianta* | 8 | B | * | + | + | * | * |
| *Neptunea arthritica* | 8 | B | – | – | + | * | * |
| *Neptunea beringiana* | 8 | B | – | + | + | – | – |
| *Neptunea borealis* | 8 | B | + | + | – | – | – |
| *Neptunea bulbacea* | 8 | B | – | – | + | + | + |
| *Neptunea communis clarki* | 8 | B | + | + | + | + | – |
| *Neptunea communis communis* | 8 | B | + | * | – | – | – |
| *Neptunea constricta* | 8 | B | – | – | + | + | * |
| *Neptunea convexa* | 8 | B | – | – | + | – | * |
| *Neptunea eulimata* | 8 | B | – | + | * | * | * |
| *Neptunea heros* | 8 | B | + | + | * | * | * |
| *Neptunea insularis* | 8 | B | – | * | + | + | + |
| *Neptunea intersculpta* | 8 | B | * | * | + | + | + |
| *Neptunea laeva* | 8 | B | – | – | + | – | – |
| *Neptunea lamellosa* | 8 | B | – | * | + | + | + |
| *Neptunea laticostata* | 8 | B | – | – | + | + | – |
| *Neptunea lyrata* | 8 | B | – | + | + | + | + |
| *Neptunea ochotense* | 8 | B | – | – | + | – | – |
| *Neptunea oncodes* | 8 | B | – | * | + | – | * |
| *Neptunea polycostata* | 8 | B | – | – | + | + | + |
| *Neptunea pribiloffensis* | 8 | B | – | + | – | – | + |
| *Neptunea tuberculata* | 8 | B | – | – | + | + | + |
| *Neptunea varicifera* | 8 | B | – | + | + | + | – |
| *Neptunea ventricosa* | 8 | B | * | + | + | – | * |
| *Neptunea vinosa* | 8 | B | – | * | + | * | + |
| *Nereis vexillosa* | 20 | B | – | * | * | * | + |
| *Nesiarchus nasutus* | 1 | MB | – | – | – | – | + |
| *Nessorhamphus danae* | 1 | M | – | – | – | – | + |
| *Nezumia proxima* | 1 | M | – | – | – | – | + |
| *Nezumia stelgidolepis* | 1 | MB | – | – | – | – | + |
| *Nibea mitsukurii* | 1 | M | – | – | – | – | + |
| *Nomeus gronovii* | 1 | M | – | – | – | – | + |
| *Notacanthus abbotti* | 1 | B | – | – | – | – | + |
| *Notacanthus chemnitzii* | 1 | MB | – | + | + | – | + |
| *Nothria conchylega* | 20 | B | * | * | + | – | + |
| *Notoscopelus caudispinosus* | 1 | M | – | – | – | – | + |
| *Notoscopelus japonicus* | 1 | MB | – | – | – | – | + |
| *Notoscopelus resplendens* | 1 | M | – | – | – | – | + |
| *Notostomus japonicus* | 5 | B | – | * | – | – | + |
| *Nototodarus hawaiiensis* | 7 | B | – | – | – | – | + |
| *Nucella heyseana* | 8 | B | – | – | * | + | * |
| *Nucella lima* | 8 | B | * | * | * | + | * |
| *Nuculana pernula* | 9 | B | * | + | + | + | * |
| *Nuttallia commoda* | 9 | B | – | – | + | + | – |
| *Nymphon hodgsoni* | 18 | B | – | – | + | * | * |
| *Occella dodecaedron* | 1 | MB | * | + | + | + | + |
| *Occella iburia* | 1 | M | – | – | – | – | + |
| *Ocenebra inornata* | 8 | B | – | – | * | + | * |
| *Octopoteuthis deletron* | 7 | MB | – | – | – | – | + |
| *Octopus californicus* | 7 | B | – | – | + | – | + |
| *Octopus conispadiceus* | 7 | MB | – | – | + | + | + |
| *Octopus longispadiceus* | 7 | B | – | – | + | * | + |
| *Octopus ochotensis* | 7 | B | – | – | + | – | + |
| *Octopus variabilis* | 7 | B | – | – | – | – | + |
| *Octopus yendoi* | 7 | B | – | + | + | * | * |
| *Ocynectes maschalis* | 1 | B | – | – | * | * | + |
| *Ocythoe tuberculata* | 7 | MB | – | + | – | – | + |
| *Odontopyxis trispinosa* | 1 | B | – | + | – | – | + |
| *Okamejei kenojei* | 1 | B | – | – | + | + | + |
| *Okutania anonycha* | 7 | MB | – | + | + | – | + |
| *Ommastrephes bartramii* | 7 | MB | – | – | + | * | + |
| *Omosudis lowii* | 1 | M | – | – | – | – | + |
| *Onchidiopsis sp.* | 8 | B | * | * | + | * | * |
| *Oncorhynchus gorbuscha* | 1 | MB | – | + | + | + | + |
| *Oncorhynchus keta* | 1 | MB | + | + | + | + | + |
| *Oncorhynchus kisutch* | 1 | MB | – | + | + | + | + |
| *Oncorhynchus masou* | 1 | MB | – | + | + | + | + |
| *Oncorhynchus mykiss* | 1 | M | – | – | + | – | + |
| *Oncorhynchus nerka* | 1 | MB | + | + | + | + | + |
| *Oncorhynchus tshawytscha* | 1 | MB | + | + | + | – | + |
| *Oneirodes bulbosus* | 1 | MB | – | + | + | – | + |
| *Oneirodes eschrichtii* | 1 | B | – | * | – | – | + |
| *Oneirodes thompsoni* | 1 | MB | – | + | + | – | + |
| *Onychoteuthis banksii* | 7 | M | – | – | – | – | + |
| *Onychoteuthis borealijaponica* | 7 | MB | – | + | + | * | + |
| Ophiolepididae gen. sp. | 13 | B | * | + | + | * | + |
| *Ophiopenia vicina* | 13 | B | – | + | + | * | + |
| *Ophiopholis aculeata* | 13 | B | * | + | + | + | + |
| *Ophiura sarsii* | 13 | B | + | + | + | + | + |
| *Opisthocentrus ocellatus* | 1 | B | – | + | + | + | + |
| *Opisthocentrus zonope* | 1 | B | – | – | + | + | – |
| *Opisthoteuthis albatrossi* | 7 | MB | – | + | + | – | + |
| *Opisthoteuthis californiana* | 7 | MB | – | + | + | – | + |
| *Opisthoteuthis depressa* | 7 | B | – | – | – | – | + |
| *Oplegnathus fasciatus* | 1 | M | – | – | – | + | * |
| *Opostomias mitsuii* | 1 | MB | – | – | – | – | + |
| *Oratosquilla oratoria* | 6 | B | – | – | – | + | – |
| *Oregonia bifurca* | 4 | B | – | * | + | – | + |
| *Oregonia gracilis* | 4 | B | – | * | + | + | + |
| *Osmerus dentex* | 1 | MB | + | + | + | + | + |
| *Ostrea sp.* | 9 | B | – | * | * | + | * |
| *Pachycheles stevensii* | 4 | B | – | – | – | + | * |
| *Pachystomias microdon* | 1 | B | – | * | – | – | + |
| *Pagurus brachiomastus* | 6 | B | – | – | – | + | * |
| *Pagurus cornutus* | 6 | B | – | + | * | – | * |
| *Pagurus gracilipes* | 6 | B | – | – | – | + | * |
| *Pagurus middendorffii* | 6 | B | – | * | + | + | * |
| *Pagurus ochotensis* | 6 | B | – | + | * | + | + |
| *Pagurus pectinatus* | 6 | B | – | – | + | + | + |
| *Pagurus pubescens* | 6 | B | – | – | + | + | + |
| *Pagurus trigonocheirus* | 6 | B | * | + | + | * | * |
| *Pagurus undosus* | 6 | B | – | + | + | * | * |
| *Pallasina barbata* | 1 | MB | * | + | + | + | + |
| *Pampus argenteus* | 1 | MB | – | – | – | + | – |
| *Pampus echinogaster* | 1 | B | – | – | * | + | – |
| *Pandalopsis coccinata* | 5 | B | – | + | + | – | + |
| *Pandalopsis dispar* | 5 | B | + | + | – | – | * |
| *Pandalopsis glabra* | 5 | B | – | + | + | – | + |
| *Pandalopsis japonicus* | 5 | MB | – | – | + | + | – |
| *Pandalopsis lamelligera* | 5 | MB | – | – | + | + | – |
| *Pandalopsis longirostris* | 5 | B | – | * | + | – | * |
| *Pandalopsis multidentatus* | 5 | B | – | + | + | + | – |
| *Pandalopsis ochotensis* | 5 | B | – | + | + | – | – |
| *Pandalopsis punctata* | 5 | B | – | – | + | – | – |
| *Pandalus borealis* | 5 | MB | + | + | + | + | + |
| *Pandalus eous* | 5 | B | + | * | + | + | * |
| *Pandalus goniurus* | 5 | MB | + | + | + | + | + |
| *Pandalus hypsinotus* | 5 | MB | – | + | + | + | + |
| *Pandalus jordani* | 5 | B | * | + | * | – | + |
| *Pandalus latirostris* | 5 | B | – | + | * | + | * |
| *Pandalus montagui* | 5 | B | * | + | – | – | + |
| *Pandalus prensor* | 5 | B | – | – | + | + | + |
| *Pandalus tridens* | 5 | B | * | + | – | – | * |
| *Panomya norvegica* | 9 | B | * | + | + | + | * |
| *Parabothus coarctatus* | 1 | B | – | – | – | – | + |
| *Paracrangon echinata* | 5 | B | – | * | + | + | + |
| *Paradorippe granulata* | 4 | B | – | – | – | + | * |
| *Paragorgia arborea* | 14 | B | – | * | + | – | + |
| *Paralichthys olivaceus* | 1 | MB | – | – | * | + | – |
| *Paraliparis dactylosus* | 1 | MB | – | + | + | – | + |
| *Paraliparis entochloris* | 1 | MB | – | – | + | – | + |
| *Paraliparis grandis* | 1 | B | – | + | + | – | + |
| *Paraliparis holomelas* | 1 | B | – | * | + | – | * |
| *Paraliparis melanobranchus* | 1 | MB | – | + | + | – | + |
| *Paraliparis rosaceus* | 1 | M | – | – | + | – | * |
| *Paraliparis ulochir* | 1 | B | – | + | – | – | – |
| *Paralithodes brevipes* | 4 | B | – | + | + | + | + |
| *Paralithodes camtschaticus* | 4 | B | – | + | + | + | + |
| *Paralithodes platypus* | 4 | B | + | + | + | + | + |
| *Paralomis multispina* | 4 | B | – | + | + | – | + |
| *Paralomis verrilli* | 4 | B | – | * | + | – | + |
| *Paramonacanthus japonicus* | 1 | M | – | – | – | – | + |
| *Paraulopus filamentosus* | 1 | B | – | – | – | – | + |
| *Parmaturus pilosus* | 1 | M | – | – | – | – | + |
| *Parophrys vetulus* | 1 | B | – | + | – | – | + |
| *Parvamussium alaskense* | 9 | B | – | * | + | + | + |
| *Pasiphaea pacifica* | 5 | MB | – | * | + | – | + |
| Patellidae gen. sp. | 8 | B | – | * | + | * | * |
| *Patiria pectinifera* | 13 | B | – | – | + | + | + |
| *Pavonaria finmarchica* | 14 | B | – | + | + | + | + |
| Penaeidae gen. sp. | 5 | B | – | * | + | * | * |
| *Pennahia argentata* | 1 | M | – | – | – | – | + |
| *Pentaceros japonicus* | 1 | MB | – | – | – | * | + |
| *Pentamera calcigera* | 12 | B | * | * | * | + | * |
| *Peprilus simillimus* | 1 | B | – | – | – | – | + |
| *Percis japonica* | 1 | MB | + | + | + | + | + |
| *Perigonimus sp.* | 14 | M | * | + | – | – | * |
| *Periphylla periphylla* | 14 | MB | – | + | + | – | + |
| Peristediidae gen. sp. | 1 | B | – | – | – | – | + |
| *Peronidia zyonoensis* | 9 | B | – | * | * | + | * |
| *Petroschmidtia albonotatus* | 1 | B | – | – | + | * | – |
| *Petroschmidtia toyamensis* | 1 | B | – | – | * | + | – |
| *Phacellophora camtschatica* | 14 | MB | – | + | + | – | + |
| *Pholidapus dybowskii* | 1 | B | – | – | + | + | + |
| *Pholidoteuthis massyae* | 7 | B | – | – | – | – | + |
| *Pholis fasciata* | 1 | B | * | + | + | * | * |
| *Pholis nebulosa* | 1 | B | – | – | – | + | * |
| *Pholis ornata* | 1 | B | – | – | – | + | – |
| *Pholis picta* | 1 | B | – | – | + | + | + |
| *Photonectes albipennis* | 1 | M | – | – | – | – | + |
| *Photonectes margarita* | 1 | M | – | – | – | – | + |
| *Photostomias guernei* | 1 | B | – | – | – | – | + |
| *Physiculus japonicus* | 1 | MB | – | – | * | – | + |
| Pinnotheridae gen. sp. | 4 | B | – | * | – | + | * |
| *Pisoides bidentatus* | 4 | B | – | – | – | + | + |
| *Platichthys stellatus* | 1 | MB | + | + | + | + | + |
| *Plectranthias kelloggi* | 1 | B | – | – | – | – | + |
| *Pleurogrammus azonus* | 1 | MB | – | – | + | + | + |
| *Pleurogrammus monopterygius* | 1 | MB | + | + | + | + | + |
| *Pleuronectes quadrituberculatus* | 1 | MB | + | + | + | + | + |
| *Pleuronichthys decurrens* | 1 | B | – | + | – | – | * |
| *Plicifusus elaeodes* | 8 | B | – | – | + | * | – |
| *Plicifusus kroyeri* | 8 | B | * | * | + | * | * |
| *Plicifusus plicatus* | 8 | B | – | + | + | + | * |
| *Plumarella longispina* | 14 | B | – | – | + | – | + |
| *Pododesmus macrochisma* | 9 | B | – | * | + | + | + |
| *Podothecus accipenserinus* | 1 | MB | + | + | + | + | + |
| *Podothecus sachi* | 1 | MB | – | – | + | + | + |
| *Podothecus sturioides* | 1 | MB | – | + | + | + | + |
| *Podothecus veternus* | 1 | MB | + | + | + | + | + |
| Pogonophora gen. sp. | 20 | B | – | – | – | + | * |
| *Polyacanthonotus challengeri* | 1 | B | – | * | + | – | + |
| *Polyipnus matsubarai* | 1 | MB | – | – | – | – | + |
| *Polymastia kurilensis* | 17 | B | + | * | * | * | * |
| *Polymastia mamillaris* | 17 | B | + | * | * | – | * |
| *Polymixia japonica* | 1 | B | – | – | – | – | + |
| *Polypera greeni* | 1 | B | – | + | – | – | * |
| *Polypera simushirae* | 1 | B | – | + | – | – | + |
| *Porichthys notatus* | 1 | B | – | – | – | – | + |
| *Poroclinus rothrocki* | 1 | B | – | + | – | – | + |
| *Porocottus camtschaticus* | 1 | B | – | – | + | – | * |
| *Porocottus japonicus* | 1 | B | – | – | * | + | – |
| *Porocottus mednius* | 1 | B | – | * | + | – | + |
| *Porocottus minutus* | 1 | B | – | – | + | – | – |
| *Porocottus quadrifilis* | 1 | M | – | + | – | – | – |
| *Porogadus sp.* | 1 | B | – | – | – | – | + |
| *Poromitra crassiceps* | 1 | MB | – | + | + | – | + |
| *Priacanthus macracanthus* | 1 | M | – | – | – | * | + |
| *Priapulus caudatus* | 20 | B | + | * | + | * | * |
| *Primnoa pacifica* | 14 | B | – | * | + | * | * |
| *Prionace glauca* | 1 | MB | – | – | + | + | + |
| *Pristigenys niphonia* | 1 | M | – | – | – | – | + |
| *Promethichthys prometheus* | 1 | MB | – | – | – | – | + |
| *Propagurus obtusifrons* | 6 | B | – | – | – | + | * |
| *Protomyctophum thompsoni* | 1 | MB | – | + | + | – | + |
| *Psenes maculatus* | 1 | M | – | – | – | – | + |
| *Psenes pellucidus* | 1 | M | – | – | – | – | + |
| *Psenopsis anomala* | 1 | M | – | – | – | – | + |
| *Psettichthys melanostictus* | 1 | B | – | – | – | – | + |
| *Pseudarchaster parelii* | 13 | B | – | + | + | + | + |
| *Pseudobathylagus milleri* | 1 | MB | – | + | + | – | + |
| *Pseudopentaceros wheeleri* | 1 | MB | – | – | – | – | + |
| *Pseudopleuronectes herzensteini* | 1 | B | – | – | + | + | + |
| *Pseudopleuronectes obscurus* | 1 | B | – | – | + | + | * |
| *Pseudopleuronectes schrenki* | 1 | B | – | – | + | + | + |
| *Pseudopleuronectes yokohomae* | 1 | B | – | – | + | + | + |
| *Pseudoscopelus altipinnis* | 1 | M | – | – | – | – | + |
| *Pseudoscopelus sagamianus* | 1 | M | – | – | – | – | + |
| *Psolus fabricii* | 12 | B | * | * | * | + | * |
| *Psolus japonicus* | 12 | B | – | – | + | + | + |
| *Psolus peronii* | 12 | B | + | * | + | + | * |
| *Psolus phantapus* | 12 | B | * | * | + | + | * |
| *Psychrolutes paradoxus* | 1 | B | – | + | + | + | + |
| *Psychrolutes phrictus* | 1 | B | – | + | + | + | + |
| *Pteraclis aesticola* | 1 | M | – | – | – | – | + |
| *Pteraclis velifera* | 1 | M | – | – | – | – | + |
| *Pteraster marsippus* | 13 | B | – | * | + | + | * |
| *Pteraster militaris* | 13 | B | – | * | + | + | * |
| *Pteraster obscurus* | 13 | B | + | + | + | + | * |
| *Pteraster tesselatus* | 13 | B | + | + | + | + | + |
| *Pteroplatytrygon violacea* | 1 | M | – | – | – | – | + |
| *Pterothrissus gissu* | 1 | M | – | – | + | * | + |
| *Pterotrachea coronata* | 8 | M | – | – | – | – | + |
| *Pterygioteuthis gemmata* | 7 | M | – | – | – | – | + |
| *Ptilichthys goodei* | 1 | MB | – | + | + | + | + |
| *Ptychogena lactea* | 14 | MB | + | + | + | + | + |
| *Pugettia quadridens* | 4 | B | – | – | * | + | * |
| *Pungitius pungitius* | 1 | MB | – | + | + | + | – |
| *Pungitius sinensis* | 1 | B | – | – | – | + | – |
| *Puzanovia sp.* | 1 | B | – | – | – | – | + |
| *Pyrosoma atlanticum* | 3 | MB | – | – | – | – | + |
| *Pyroteuthis addolux* | 7 | M | – | – | – | – | + |
| *Pyroteuthis margaritifera* | 7 | M | – | – | – | – | + |
| *Pyrulofusus deformis* | 8 | B | + | + | + | – | + |
| *Pyrulofusus dexius* | 8 | B | – | * | – | – | + |
| *Radulinopsis derjavini* | 1 | B | – | – | – | + | + |
| *Raja binoculata* | 1 | B | – | + | – | – | + |
| *Raja inornata* | 1 | B | – | – | – | – | + |
| *Raja pulchra* | 1 | B | – | – | + | + | + |
| *Raja rhina* | 1 | B | – | + | – | – | + |
| *Raja stellulata* | 1 | B | – | + | – | – | + |
| *Rapana sp.* | 8 | B | – | – | – | + | * |
| *Rastrinus scutiger* | 1 | B | – | * | – | + | * |
| *Reinhardtius hippoglossoides* | 1 | MB | + | + | + | * | + |
| *Remora remora* | 1 | M | – | – | – | – | + |
| *Retepora sp.* | 14 | B | – | * | – | – | + |
| *Rexea solandri* | 1 | M | – | – | – | – | + |
| *Rhinochimaera pacifica* | 1 | MB | – | – | – | – | + |
| *Rhinoliparis barbulifer* | 1 | B | – | * | + | – | * |
| *Rhinoraja longicauda* | 1 | B | – | – | * | – | + |
| *Rhinoraja taranetzi* | 1 | MB | – | + | + | – | + |
| *Rhodymenichthys dolichogaster* | 1 | MB | * | + | * | + | * |
| *Rhopilema esculentum* | 14 | B | – | – | – | + | * |
| *Rocinela maculata* | 6 | B | – | * | + | * | + |
| *Rondeletia loricata* | 1 | MB | – | – | * | – | + |
| *Ronquilus jordani* | 1 | B | – | + | – | – | + |
| *Rossia pacifica* | 7 | MB | – | + | + | + | + |
| *Rouleina sp.* | 1 | B | – | – | – | – | + |
| *Ruvettus pretiosus* | 1 | MB | – | – | – | – | + |
| *Sabinea septemcarinata* | 5 | B | + | – | – | – | – |
| *Sagamichthys abei* | 1 | MB | – | + | + | – | + |
| *Salangichthys microdon* | 1 | MB | – | – | – | + | + |
| *Salpa aspera* | 3 | M | – | – | – | – | + |
| *Salpa fusiformis* | 3 | M | – | – | – | – | + |
| *Salpa maxima* | 3 | M | – | – | – | – | + |
| *Salvelinus leucomaenis* | 1 | MB | – | + | + | + | + |
| *Salvelinus malma* | 1 | MB | + | + | + | + | + |
| *Sandalops melancholicus* | 7 | M | – | – | – | – | + |
| *Sarda orientalis* | 1 | M | – | – | – | * | + |
| *Sardinops melanosticta* | 1 | MB | – | * | + | + | + |
| *Sarritor frenatus* | 1 | MB | – | + | + | + | + |
| *Sarritor knipowitschi* | 1 | B | – | – | * | + | * |
| *Sarritor leptorhynchus* | 1 | MB | + | + | + | + | + |
| *Scaeurgus unicirrhus* | 7 | B | – | – | – | – | + |
| *Scaphechinus griseus* | 11 | B | – | – | – | + | – |
| *Scelidotoma gigas* | 8 | B | – | – | – | + | * |
| *Schizoplax brandtii* | 10 | B | – | * | – | – | + |
| *Sclerocrangon boreas* | 5 | MB | + | + | + | + | + |
| *Sclerocrangon derjugini* | 5 | B | – | – | + | – | + |
| *Sclerocrangon salebrosa* | 5 | MB | * | + | + | + | + |
| *Sclerocrangon sharpi* | 5 | B | – | – | – | + | – |
| *Scomber australasicus* | 1 | M | – | – | – | – | + |
| *Scomber japonicus* | 1 | MB | – | – | + | + | + |
| *Scombrolabrax heterolepis* | 1 | M | – | – | – | – | + |
| Scombropidae gen. sp. | 1 | M | – | – | – | – | + |
| *Scopelengys tristis* | 1 | M | – | + | – | – | – |
| *Scopeloberyx opisthopterus* | 1 | B | – | – | – | – | + |
| *Scopeloberyx robustus* | 1 | B | – | – | – | – | + |
| *Scopelosaurus adleri* | 1 | M | – | + | + | – | + |
| *Scopelosaurus harryi* | 1 | MB | – | + | + | – | + |
| Scorpaenidae gen. sp. | 1 | MB | – | + | + | + | + |
| *Sculptolithodes derjugini* | 4 | B | – | * | * | + | – |
| *Searsia sp.* | 1 | B | – | + | – | – | – |
| *Sebastes aleutianus* | 1 | MB | – | + | – | – | + |
| *Sebastes alutus* | 1 | MB | – | + | + | – | + |
| *Sebastes aurora* | 1 | B | – | – | – | – | + |
| *Sebastes babcocki* | 1 | B | – | – | – | – | + |
| *Sebastes baramenuke* | 1 | B | – | – | – | + | + |
| *Sebastes borealis* | 1 | MB | – | + | + | – | + |
| *Sebastes brevispinis* | 1 | B | – | – | – | – | + |
| *Sebastes chlorostictus* | 1 | B | – | – | – | – | + |
| *Sebastes ciliatus* | 1 | MB | – | + | – | – | + |
| *Sebastes crameri* | 1 | B | – | – | – | – | + |
| *Sebastes diploproa* | 1 | B | – | – | – | – | + |
| *Sebastes elongatus* | 1 | B | – | – | – | – | + |
| *Sebastes entomelas* | 1 | MB | – | – | – | – | + |
| *Sebastes flavidus* | 1 | MB | – | – | – | – | + |
| *Sebastes glaucus* | 1 | MB | – | + | + | + | + |
| *Sebastes goodei* | 1 | B | – | – | – | – | + |
| *Sebastes helvomaculatus* | 1 | MB | – | – | – | – | + |
| *Sebastes iracundus* | 1 | MB | – | – | – | * | + |
| *Sebastes jordani* | 1 | MB | – | – | – | – | + |
| *Sebastes levis* | 1 | B | – | – | – | – | + |
| *Sebastes melanostomus* | 1 | B | – | + | – | – | + |
| *Sebastes minor* | 1 | MB | – | + | + | + | + |
| *Sebastes owstoni* | 1 | MB | – | – | + | + | + |
| *Sebastes paucispinis* | 1 | MB | – | – | – | – | + |
| *Sebastes pinniger* | 1 | B | – | – | – | – | + |
| *Sebastes polyspinis* | 1 | MB | – | + | – | – | + |
| *Sebastes proriger* | 1 | B | – | – | – | – | + |
| *Sebastes reedi* | 1 | MB | – | – | – | – | + |
| *Sebastes ruberrimus* | 1 | B | – | – | – | – | + |
| *Sebastes rubrivinctus* | 1 | B | – | – | – | – | + |
| *Sebastes rufus* | 1 | B | – | – | – | – | + |
| *Sebastes saxicola* | 1 | B | – | – | – | – | + |
| *Sebastes schlegelii* | 1 | MB | – | – | + | + | + |
| *Sebastes semicinctus* | 1 | B | – | – | – | – | + |
| *Sebastes steindachneri* | 1 | MB | – | – | + | + | + |
| *Sebastes taczanowskii* | 1 | MB | – | – | + | + | + |
| *Sebastes trivittatus* | 1 | MB | – | – | + | + | + |
| *Sebastes variegatus* | 1 | B | – | + | – | – | + |
| *Sebastes wakiyai* | 1 | B | – | – | + | * | – |
| *Sebastes wilsoni* | 1 | B | – | – | – | – | + |
| *Sebastes zacentrus* | 1 | MB | – | – | – | – | + |
| *Sebastolobus alascanus* | 1 | MB | – | + | + | – | + |
| *Sebastolobus macrochir* | 1 | MB | – | + | + | – | + |
| *Selar crumenophthalmus* | 1 | B | – | – | – | + | – |
| *Selaroides leptolepis* | 1 | M | – | – | – | – | + |
| *Semibalanus balanoides* | 6 | B | – | – | + | + | – |
| *Semisuberites cribrosa* | 17 | B | + | * | * | * | * |
| *Sepiola birostrata* | 7 | MB | – | + | – | + | + |
| *Sergestes atlanticus* | 5 | M | – | – | + | – | + |
| *Seriola lalandi* | 1 | M | – | – | – | + | + |
| *Seriola quinqueradiata* | 1 | MB | – | – | – | + | + |
| *Seriola rivoliana* | 1 | M | – | – | – | – | + |
| *Serpula vermicularis* | 20 | B | – | – | + | * | + |
| *Serripes groenlandicus* | 9 | B | + | + | + | + | + |
| *Serripes laperousii* | 9 | B | * | + | + | * | * |
| *Serrivomer sector* | 1 | M | – | – | – | – | + |
| *Sertularia robusta* | 14 | B | – | * | * | – | + |
| *Setarches guentheri* | 1 | B | – | – | – | – | + |
| *Sigmops elongatus* | 1 | MB | – | – | – | – | + |
| *Sigmops gracilis* | 1 | MB | – | + | + | – | + |
| *Siliqua alta* | 9 | B | + | * | + | * | * |
| *Simenchelys parasitica* | 1 | MB | – | – | – | – | + |
| *Soestia zonaria* | 3 | M | – | – | – | – | + |
| *Solaster dawsoni* | 13 | B | + | * | * | + | * |
| *Solaster endeca* | 13 | B | – | * | – | + | * |
| *Solaster intermedius* | 13 | B | – | – | – | + | – |
| *Solaster pacificus* | 13 | B | – | – | + | + | + |
| *Solaster paxillatus* | 13 | B | – | + | – | + | * |
| *Solaster stimpsoni* | 13 | B | – | * | * | + | * |
| *Soldatovia polyactocephala* | 1 | B | – | – | + | + | * |
| *Solea sp.* | 1 | B | – | – | – | + | – |
| *Solen krusensterni* | 9 | B | – | – | * | + | * |
| *Somniosus pacificus* | 1 | MB | * | + | + | – | + |
| *Sphoeroides pachygaster* | 1 | M | – | – | – | * | + |
| *Spinther hystrix* | 20 | B | – | – | + | – | * |
| *Spirontocaris arcuata* | 5 | B | * | + | + | + | * |
| *Spirontocaris intermedia* | 5 | B | * | * | + | – | * |
| *Spirontocaris liljeborgii* | 5 | B | – | + | – | – | * |
| *Spirontocaris murdochi* | 5 | B | + | + | + | + | * |
| *Spirontocaris ochotensis* | 5 | B | – | * | + | + | * |
| *Spirontocaris phippsii* | 5 | B | * | + | * | + | * |
| *Spirontocaris spinus* | 5 | MB | * | + | + | + | * |
| *Spisula sachalinensis* | 9 | B | – | – | + | + | + |
| *Squalogadus modificatus* | 1 | MB | – | – | – | – | + |
| *Squaloliparis dentatus* | 1 | MB | – | – | + | – | + |
| *Squalus blainville* | 1 | B | – | – | – | – | + |
| *Squalus brevirostris* | 1 | B | – | – | – | + | – |
| *Squalus mitsukurii* | 1 | MB | – | – | – | – | + |
| *Squalus suckleyi* | 1 | MB | – | + | + | + | + |
| *Staurostoma mertensii* | 14 | M | * | + | – | – | * |
| *Stegophiura brachyactis* | 13 | B | – | – | * | + | * |
| *Stegophiura nodosa* | 13 | B | * | * | + | * | * |
| *Stegophiura ponderosa* | 13 | B | – | – | + | – | + |
| *Stelgistrum beringianum* | 1 | MB | – | + | – | – | + |
| *Stelgistrum concinnum* | 1 | B | – | + | – | – | – |
| *Stelgistrum stejnegeri* | 1 | B | – | – | + | + | + |
| *Stemonosudis sp.* | 1 | M | – | – | – | – | + |
| *Stenobrachius leucopsarus* | 1 | MB | – | + | + | – | + |
| *Stenobrachius nannochir* | 1 | MB | – | + | + | – | + |
| *Stephanolepis cirrhifer* | 1 | MB | – | – | + | + | * |
| *Stereolepis gigas* | 1 | B | – | – | – | + | – |
| *Sternoptyx diaphana* | 1 | MB | – | – | – | – | + |
| *Sternoptyx obscura* | 1 | M | – | – | – | – | + |
| *Sternoptyx pseudobscura* | 1 | MB | – | – | – | – | + |
| *Sthenoteuthis oualaniensis* | 7 | M | – | – | – | – | + |
| *Stichaeopsis epallax* | 1 | B | – | – | + | + | * |
| *Stichaeopsis nana* | 1 | B | – | – | + | * | * |
| *Stichaeopsis nevelskoi* | 1 | MB | – | – | + | + | * |
| *Stichaeus grigorjewi* | 1 | MB | – | – | + | + | + |
| *Stichaeus nozawae* | 1 | B | – | – | + | + | + |
| *Stichaeus ochriamkini* | 1 | B | – | * | + | + | * |
| *Stichaeus punctatus* | 1 | MB | + | + | + | + | + |
| *Stomias affinis* | 1 | M | – | – | – | – | + |
| *Stomias longibarbatus* | 1 | M | – | – | – | – | + |
| *Stomias nebulosus* | 1 | M | – | – | – | – | + |
| *Strongylocentrotus droebachiensis* | 11 | B | * | * | + | – | * |
| *Strongylocentrotus intermedius* | 11 | B | – | – | + | + | * |
| *Strongylocentrotus pallidus* | 11 | B | + | + | + | + | + |
| *Strongylocentrotus polyacanthus* | 11 | B | – | * | + | – | * |
| *Styela clava* | 3 | B | – | * | – | + | * |
| *Suberites carnosus* | 17 | B | + | – | – | * | * |
| *Suberites domuncula* | 17 | B | – | * | + | + | + |
| *Suberites ficus* | 17 | B | + | + | – | – | * |
| *Swiftopecten swifti* | 9 | B | – | – | + | + | + |
| *Symbolophorus californiensis* | 1 | MB | – | + | + | – | + |
| *Symbolophorus evermanni* | 1 | M | – | – | + | – | + |
| *Symplectoscyphus tricuspidatus* | 14 | B | – | * | + | * | * |
| *Synallactes nozawai* | 12 | B | – | * | + | + | * |
| *Synaphobranchus kaupii* | 1 | MB | – | – | + | – | + |
| *Synchiropus altivelis* | 1 | B | – | – | – | – | + |
| *Syngnathus schlegeli* | 1 | M | – | – | – | + | * |
| *Tactostoma macropus* | 1 | MB | – | + | + | – | + |
| *Takifugu chinensis* | 1 | MB | – | – | – | + | – |
| *Takifugu niphobles* | 1 | MB | – | – | * | + | – |
| *Takifugu pardalis* | 1 | B | – | – | – | + | – |
| *Takifugu porphyreus* | 1 | MB | – | – | + | + | – |
| *Takifugu rubripes* | 1 | MB | – | – | * | + | * |
| *Takifugu stictonotus* | 1 | M | – | – | – | + | + |
| *Takifugu vermicularis* | 1 | MB | – | – | – | + | + |
| *Takifugu xanthopterus* | 1 | MB | – | – | + | + | – |
| *Taningia danae* | 7 | M | – | – | – | – | + |
| *Taonius borealis* | 7 | MB | – | + | + | – | + |
| *Taractes asper* | 1 | M | – | – | – | – | + |
| *Taractichthys steindachneri* | 1 | M | – | – | – | – | + |
| *Tarletonbeania crenularis* | 1 | MB | – | + | + | – | + |
| *Taurocottus bergii* | 1 | B | – | – | + | + | + |
| *Tecticeps renoculis* | 6 | B | – | * | * | – | + |
| *Tellina lutea* | 9 | B | * | * | + | * | * |
| *Telmessus cheiragonus* | 4 | B | * | + | + | + | + |
| *Tetragonurus atlanticus* | 1 | M | – | – | – | – | + |
| *Tetragonurus cuvieri* | 1 | MB | – | – | + | – | + |
| *Tetrosomus concatenatus* | 1 | M | – | – | – | – | + |
| *Tetrosomus gibbosus* | 1 | M | – | – | – | – | + |
| *Thalassenchelys coheni* | 1 | M | – | – | – | – | + |
| *Thalassobathia pelagica* | 1 | M | – | + | + | – | – |
| *Thaleichthys pacificus* | 1 | B | – | + | – | – | + |
| *Thalia sp.* | 3 | M | – | – | – | + | * |
| *Thamnaconus modestus* | 1 | MB | – | – | – | + | + |
| *Theragra chalcogramma* | 1 | MB | + | + | + | + | + |
| *Thetys vagina* | 3 | MB | – | – | – | + | + |
| *Thracia sp.* | 9 | B | * | * | * | + | * |
| *Thuiaria thuja* | 14 | B | – | * | * | * | + |
| *Thunnus alalunga* | 1 | M | – | – | – | – | + |
| *Thunnus obesus* | 1 | M | – | – | – | – | + |
| *Thunnus orientalis* | 1 | M | – | – | + | – | + |
| *Thysanoteuthis rhombus* | 7 | M | – | – | – | * | + |
| *Tilesina gibbosa* | 1 | MB | – | – | + | + | + |
| *Tima saghalinensis* | 14 | MB | + | + | + | – | + |
| *Todarodes pacificus* | 7 | MB | – | + | + | + | + |
| *Torpedo californica* | 1 | B | – | – | – | – | + |
| *Trachipterus ishikawae* | 1 | M | – | – | – | + | + |
| *Trachipterus trachypterus* | 1 | MB | – | – | – | – | + |
| *Trachurus japonicus* | 1 | MB | – | – | – | + | + |
| *Trachurus symmetricus* | 1 | MB | – | – | – | – | + |
| *Tremoctopus gelatus* | 7 | M | – | – | – | – | + |
| *Tribolodon brandtii* | 1 | B | – | – | – | + | – |
| *Tribolodon hakonensis* | 1 | B | – | – | – | + | – |
| *Trichiurus japonicus* | 1 | M | – | – | – | * | + |
| *Trichiurus lepturus* | 1 | M | – | – | – | + | + |
| *Trichocottus brashnikovi* | 1 | MB | * | + | + | + | – |
| *Trichodon trichodon* | 1 | MB | – | + | + | + | + |
| *Trichotropis bicarinata* | 8 | B | * | * | + | * | + |
| *Triglops forficatus* | 1 | MB | – | + | + | – | + |
| *Triglops jordani* | 1 | MB | – | + | + | + | + |
| *Triglops macellus* | 1 | B | – | + | – | – | – |
| *Triglops metopias* | 1 | B | – | + | * | * | – |
| *Triglops pingelii* | 1 | MB | + | + | + | + | + |
| *Triglops scepticus* | 1 | MB | – | + | + | + | + |
| *Triodon sp.* | 1 | M | – | – | – | – | + |
| *Triphoturus nigrescens* | 1 | M | – | – | – | – | + |
| *Tritodynamia rathbunae* | 4 | B | – | – | – | + | * |
| *Tritonia diomedea* | 8 | MB | – | + | + | + | * |
| *Trophodiscus uber* | 13 | B | – | – | + | + | + |
| *Turritella fortilirata* | 8 | B | – | – | * | + | * |
| *Tylosurus acus melanotus* | 1 | M | – | – | – | – | + |
| Uranoscopidae gen. sp. | 1 | M | – | – | – | – | + |
| *Urechis sp.* | 20 | B | – | * | * | + | * |
| *Urolophus aurantiacus* | 1 | M | – | – | – | – | + |
| *Velutina bartschi* | 8 | B | – | – | – | + | – |
| *Verasper sp.* | 1 | B | – | – | – | + | – |
| *Vinciguerria nimbaria* | 1 | M | – | – | – | – | + |
| Virgulariidae gen. sp. | 14 | B | – | * | * | * | + |
| *Volutharpa ampullacea* | 8 | B | – | * | – | + | * |
| *Volutopsius castaneus* | 8 | B | * | + | + | + | + |
| *Volutopsius fragilis* | 8 | B | + | + | – | – | * |
| *Volutopsius middendorffi* | 8 | B | – | + | – | + | * |
| *Watasenia scintillans* | 7 | MB | – | – | + | + | + |
| *Winteria telescopa* | 1 | M | – | – | – | – | + |
| *Xeneretmus latifrons* | 1 | B | – | – | – | – | + |
| *Xeneretmus triacanthus* | 1 | B | – | – | – | – | + |
| Xenocongridae gen. sp. | 1 | B | – | – | – | – | + |
| *Xenolepidichthys dalgleishi* | 1 | M | – | – | – | – | + |
| *Xenolumpenus longipterus* | 1 | B | – | – | * | + | * |
| *Xiphias gladius* | 1 | M | – | – | + | * | + |
| *Xyelacyba sp.* | 1 | B | – | – | – | – | + |
| *Yoldia hyperborea* | 9 | B | + | + | + | * | * |
| *Yoldia seminuda* | 9 | B | * | * | * | + | * |
| *Zalembius rosaceus* | 1 | B | – | – | – | – | + |
| *Zameus squamulosus* | 1 | M | – | – | – | – | + |
| *Zaniolepis latipinnis* | 1 | B | – | – | – | – | + |
| *Zaprora silenus* | 1 | MB | – | + | + | – | + |
| *Zenion japonicum* | 1 | B | – | – | – | * | + |
| *Zenopsis nebulosa* | 1 | MB | – | – | – | * | + |
| *Zesticelus bathybius* | 1 | B | – | – | + | – | * |
| *Zesticelus profundorum* | 1 | B | – | * | + | – | + |
| *Zestichthys tanakai* | 1 | M | – | – | – | – | + |
| *Zeus faber* | 1 | M | – | – | – | – | + |
| *Zoarces andriashevi* | 1 | B | – | – | + | – | – |
| *Zoarces elongatus* | 1 | MB | – | – | + | + | * |
| *Zu cristatus* | 1 | M | – | – | – | – | + |
